# Discovery, optimization, and evaluation of non-bile acid FXR/TGR5 dual agonists

**DOI:** 10.1038/s41598-021-88493-0

**Published:** 2021-04-28

**Authors:** Sachiho Miyata, Yuji Kawashima, Miku Sakai, Masaya Matsubayashi, Keisuke Motoki, Yui Miyajima, Yousuke Watanabe, Noriko Chikamatsu, Tetsuya Taniguchi, Ryukou Tokuyama

**Affiliations:** 1 Research Laboratory 1, FUJI YAKUHIN. CO., LTD, 1-32-3, Nishi-Omiya, Nishi-ku, Saitama City, Saitama Japan; 2 Research Laboratory 2, FUJI YAKUHIN. CO., LTD, Nishi-ku, Iida-Shinden, Saitama City, Saitama 636-1 Japan

**Keywords:** Chemical biology, Drug discovery, Medical research

## Abstract

Although several potent bile acid Farnesoid X receptor (FXR) and Takeda G-protein-coupled receptor 5 (TGR5, GPBAR1) dual agonists such as INT-767 have been reported, no non-bile acid FXR/TGR5 dual agonist has been investigated to date. Therefore, we attempted to discover potent non-bile acid FXR/TGR5 dual agonists and identified some non-bile acid FXR/TGR5 dual agonists, such as isonicotinamide derivatives in vitro assay. Compound **20p** was evaluated in C57BL/6J mice, that were administered a choline-deficient, L-amino acid-defined, high-fat diet (CDAHFD) consisting of 60 kcal% fat and 0.1% methionine by weight for one week. Compound **20p** dose-dependently induced small heterodimer partner (SHP) mRNA and decreased cytochrome P450 7A1 (CYP7A1) in the liver at 10 and 30 mg/kg, respectively, which were used as FXR agonist markers. Compound **20p** significantly increased the plasma levels of GLP-1 as a TGR5 agonist, and a high concentration of GLP-1 lowered blood glucose levels. We confirmed that compound **20p** was a non-bile acid FXR/TGR5 dual agonist.

## Introduction

Type 2 diabetes and nonalcoholic fatty liver disease (NAFLD) are serious hepatic disease events^1^. Metabolic syndromes, including insulin resistance, hypertension, dyslipidemia, and obesity, are closely associated with the pathogenesis of NAFLD^2^. Approximately 20–30% NAFLD patients develop nonalcoholic steatohepatitis (NASH)^3^, which can lead to cirrhosis and hepatocellular carcinoma^4,5^.

The farnesoid X receptor (FXR, NR1H4), a member of the nuclear receptor (NR) superfamily, is a ligand-induced transcription factor^6,7^. The expression levels of FXR are high in the liver, intestine, kidney, and adrenal glands^8–10^. The bile acid (BA), chenodeoxycholic acid (CDCA), is an endogenous ligand against FXR. BAs play key roles in regulating cholesterol and bile acid homeostasis^11^. Activation of FXR in the liver induces a downregulation of CYP7A1 gene transcription. CYP7A1 regulates the conversion of free cholesterol to BAs, and the downregulation of CYP7A1 upregulates a small heterodimer partner (SHP)^12^.

Obeticholic acid (OCA, INT-747, **2**) is the first steroidal FXR agonist. In the combination with ursodeoxycholic acid, it reduces alkaline phosphatase levels in patients with primary biliary cirrhosis^13^. A proof-of-concept study reported that OCA significantly improves insulin sensitivity and reduces liver inflammation and fibrosis markers, such as alanine aminotransferase (ALT) and enhanced liver fibrosis score, in patients with type 2 diabetes and NAFLD^14^. OCA can also be used for the treatment of patients with NASH. It has been reported that 25 mg/day of the FXR ligand, OCA, improves NAFLD activity score (NAS) and fibrosis status in the livers of noncirrhotic, nonalcoholic steatohepatitis patients. However, OCA causes pruritus as a clinical adverse event^15^.

Several potent and selective FXR agonists^16–18^, such as GW4064 (**4**), have been reported. GW4064 is a selective nonsteroidal FXR agonist^19^ that significantly reduces free fatty acid and triglyceride levels in db/db mice^20^.

Takeda G-protein-coupled receptor 5 (TGR5, GPBAR) is a class A G-protein-coupled receptor (GPCR) that is expressed in the liver, skeletal muscle, intestine, and brown adipose tissues^21^. Activation of TGR5 by BA stimulates glucagon-like peptide-1 (GLP-1) secretion from intestinal enteroendocrine L cells by increasing intracellular cAMP concentration^22–24^. A TGR5 agonist reduces lipopolysaccharide (LPS)-induced release of proinflammatory cytokines such as tumor necrosis factor alpha (TNF-α), interleukin (IL)-1, IL-6, and IL-8. Thus, TGR5 agonists may have broad therapeutic applications, from the treatment of metabolic disorders to liver inflammatory diseases^25^.

INT-767 (**3**), a steroidal FXR/TGR5 dual agonist, decreases serum cholesterol and triglyceride levels in diabetic db/db mice and streptozotocin-induced type 1 diabetes model mouse^26^. Moreover, INT-767 reduces the production of inflammatory markers, such as IL-1beta, cytokine, and TNF-α, in diabetic db/db mice and apolipoprotein E-deficient mice^27,28^. Treatment with INT-767 for 16 weeks significantly improves histological parameters as compared to OCA treatment. Furthermore, INT-767 leads to a significant reduction in NAS and fibrosis in ob/ob NASH mice model as compared to OCA^29^.

Crystallographic studies have enabled a clear understanding of the interaction between FXR agonists and the ligand binding domain (LBD), making FXR an attractive target. A cocrystal structure of GW4064 with the active site of FXR protein has been reported (PDB code 4DCT)^30^. The isoxazole moiety of GW4064 interacts with Trp454 and His447 in helix 11, forming an edge-to-face stacking interaction with Trp469 located in helix 12 (AF2). The carboxylic acid of GW4064 forms an electrostatic interaction with Arg331 in helix 5. A cocrystal structure of compound **7** with the active site of FXR has been reported (PDB code 1OSH). However, the insertion regions of 15 residues between helices 1 and 3 are completely disordered^31^. The interactions between FXR LBD and compound **7** can be divided into two sets. The first set of interactions stabilizes the position of hexyl ring, which contacts Ile339 and Leu344. The second set comprises a biaryl ring and dimethylamine group. The dimethylamine group interacts with Trp454. The side chain of Trp454 is shifted by the dimethylamine group as compared to PDB code 3DCT. The biaryl group stabilizes His447 and Trp469.

**Figure 1:**
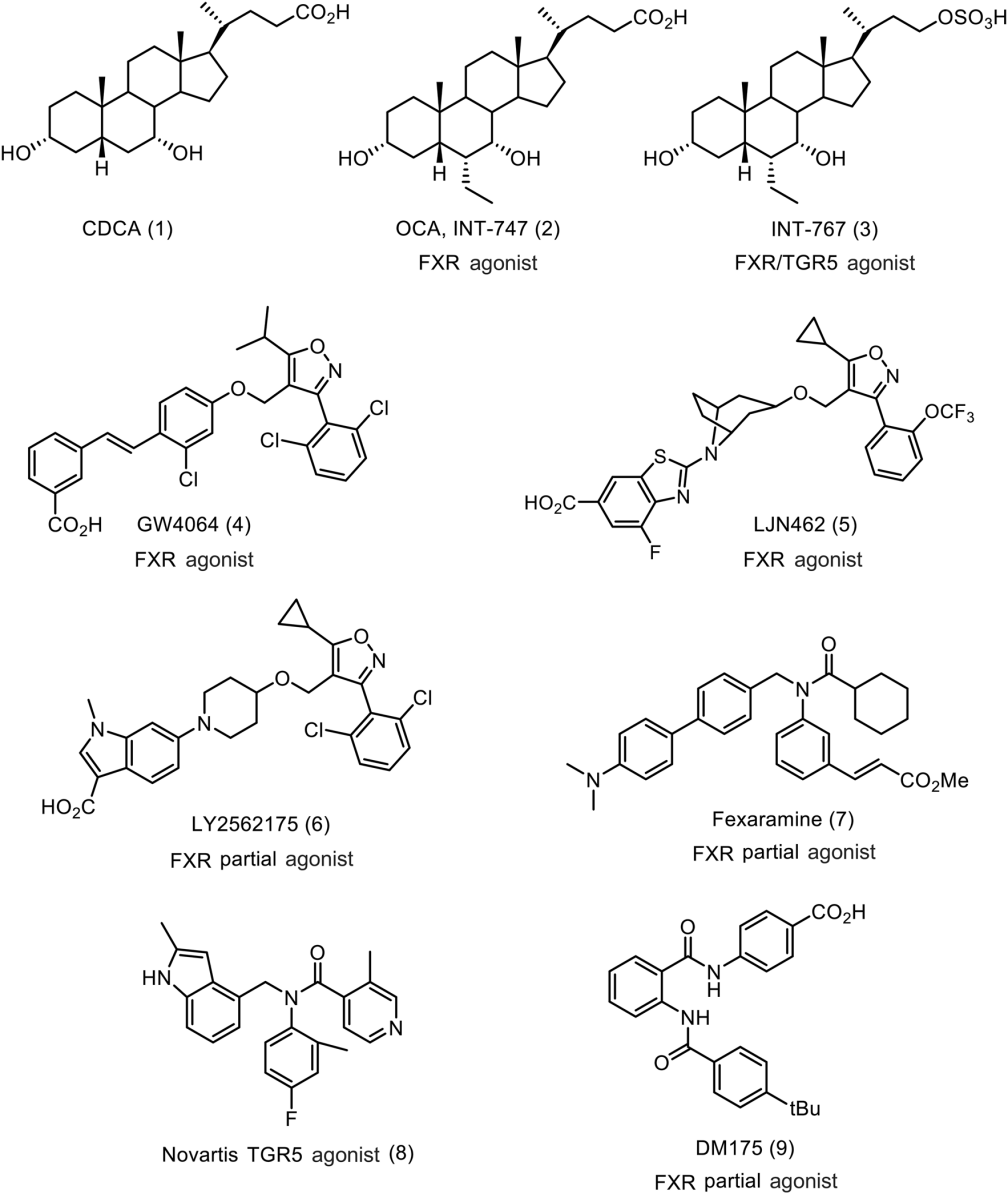
FXR and TGR5 ligands.

Several non-bile acid TGR5 agonists have been reported in literature^32^. An isonicotinamide compound has been identified as a non-bile acid TGR5 agonist (**8**, Fig. 1)^33,34^. This compound inhibits TGR5-dependent LPS-induced TNF-α and IL-12 release in mice. These results illustrate the important regulatory roles of TGR5 agonists in controlling inflammation.

Several potent bile acid FXR/TGR5 dual agonists have been reported so far, but no non-bile acid FXR/TGR5 dual agonist has been investigated to date^16^. Therefore, in this study, we attempted to discover potent non-bile acid FXR/TGR5 dual agonists. This study reports our efforts to develop a non-bile acid FXR/TGR5 dual agonists and investigate its in vitro and in vivo activities.

## Results

Fexaramine (compound **7**) was reported as a specific and potent partial agonist against FXR with no significant agonist activity against TGR5^31^. The indole derivative **8** is a TGR5 agonist (Fig. 1) with no agonist or antagonist activity against FXR^33^.

Presently, two categories of FXR and TGR5 agonists exist. One is structurally based on steroidal scaffolds, including bile acids, such as cholic acid and lithocholic acid, and semisynthetic steroid derivatives, such as OCA, INT-767, and INT-777, which are FXR and FXR/TGR5 dual or selective TGR5 agonists^16^. The other category is small molecules. Isoxazole and compound **7** derivatives are small-molecule FXR agonists^18^, and isoxazole, pyridine, and isonicotinamide derivatives are small molecule TGR5 agonists^32,33^. We observed that compound **7** showed some structural similarities to isonicotinamide derivative **8**. The isonicotinamide group of **8** is linked to the cyclohexyl group of compound **7**, and both compounds have an *N*-benzyl framework. This observation prompted us to synthesize a few chimera compounds between compound **7** and isonicotinamide **8** and to evaluate their FXR/TGR5 dual agonist activities.

To evaluate direct agonist activities against FXR, test compounds were evaluated using a CHO cell-based FXR assay by Indigo Biosciences (State College, PA), in which cells were transfected with an FXR luciferase reporter system. Cells were incubated with the test compounds for 22 h, and potency was assessed using a multi-mode microplate reader. OCA **2** was used as the reference compound for 1 µM, which we set as 100% FXR activation^35,36^.

TGR5 agonist activity was evaluated by transfecting CHO cells with TGR5 using the Discover X expression kit (cAMP Hunter eXpress GPCR Assay, Discover X Fremont, CA). Cells were incubated with test compounds for 0.5 h, and intracellular cAMP levels were measured using the multi-mode microplate reader. The response to 10 µM INT-767 **3** was set as 100% TGR5 activation^37^.

**Figure 2:**
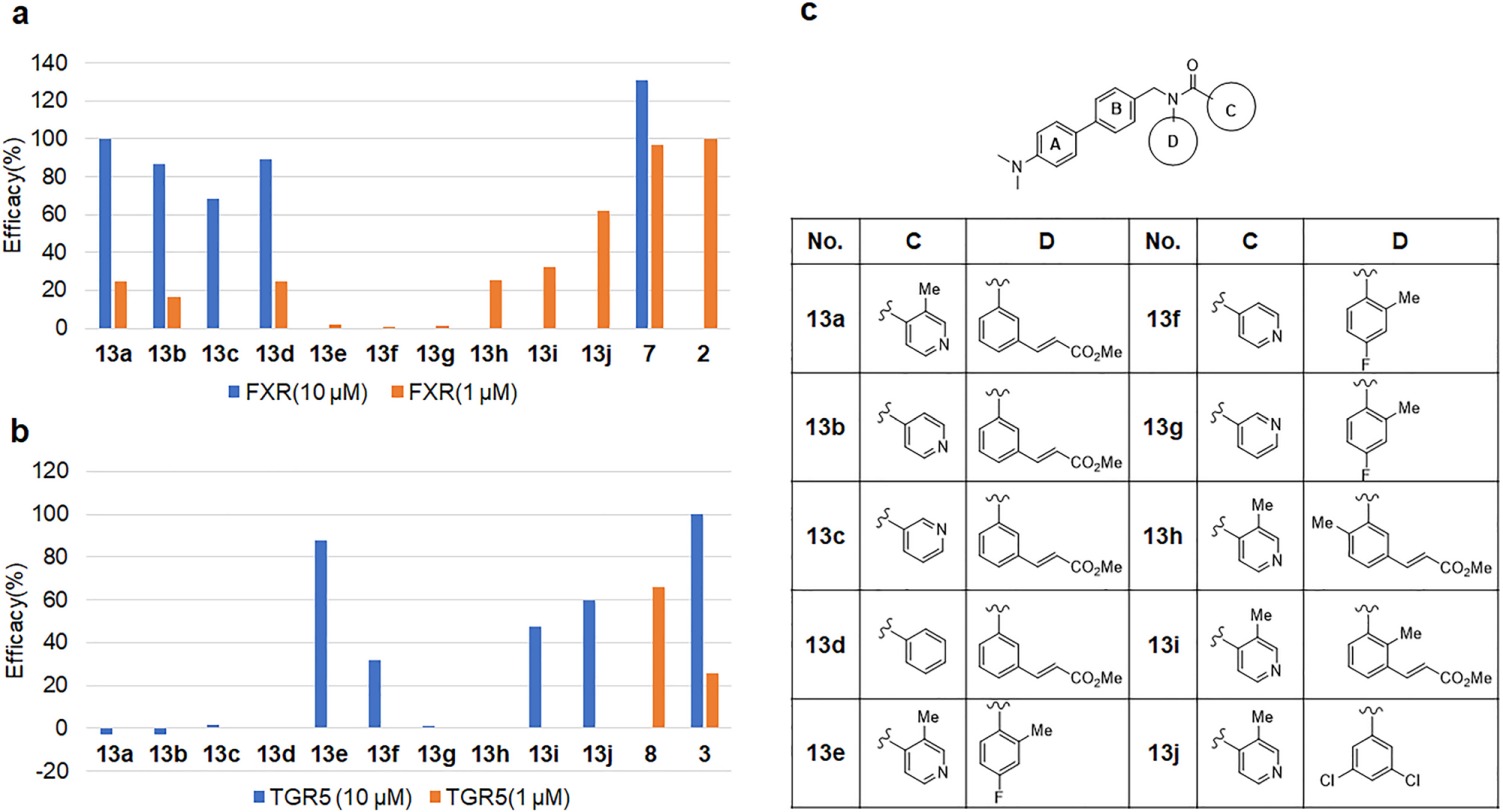
(**a**) FXR agonist acitivity of 4-dimethylamino biphenyl derivatives **13a**-**13j**. Efficacy (%); % vs OCA 1 µM as 100%, OCA; 100% (1 µM), 13.3% (0.1 µM); (**b**) TGR5 agonist activity of 4-dimethylamino biphenyl derivatives **13a**-**13j**. Efficacy (%); % vs INT-767 10 µM as 100%, INT-767 100% (10 µM), 25.5% (1 µM), 1.7% (0.1 µM). (**c**) Structures of 4-dimethylamino biphenyl derivatives **13a**-**13j**.

Compound **7** was divided into four fragments, dimethylaminophenyl group (A), center phenyl linker (B), cyclohexyl group (C), and aniline group (D), to explore the structure activity relationship (SAR). A chimera strategy between compound **7** and isonicotinamide compound **8** enabled the modulation of cyclohexyl (C) and aniline (D) groups to rapidly assess the C/D region of molecule. To investigate the activities of some chimera compounds between compound **7** and isonicotinamide compound **8** C/D phenyl ring, we synthesized compounds **13a**-**13j**, as shown in Fig. 2. Compound **7** was synthesized and optimized to incorporate a 3-methyl acrylate moiety for potent FXR agonist activity^38^. The 2-methyl isonicotinamide group is important for TGR5 agonist activity^33,34^, and ortho-substituted anilides, such as 2-methyl-4-fluoroanilide, in the center aniline group exhibit sub-nanomolar TGR5 agonist activities in the cAMP assay^33^.

Compound **13a** showed a strong FXR agonist activity with full efficacy while compounds **13b** and **13c** significantly decreased the FXR activity. The methyl group at 2-position and the position of nitrogen in C region are important for FXR agonist activity. Unfortunately, although a methyl acrylate moiety at the meta position in D region showed a potent FXR agonist activity, compounds **13a** and **13b** were inactive against TGR5. Compound **13e**, which is a similar type of compound **8**, showed a strong TGR5 agonist activity. Compounds **13f** and **13g**, however, exhibited decreased TGR5 activities. These results mimic the following SARs of Novartis compounds^33,34^: (1) the position of methyl group is important and (2) a pyridine isomer leads to a complete activity loss in the C region. To explore the disubstituted analogs in D region, we investigated 2, 3-, 2, 5-, and 3, 5-disubstituted analogs. Compound **13h** decreased TGR5 potency; however, **13i** showed a weak FXR/TGR5 dual agonist activity. The methyl acrylate group plays a critical role in FXR agonist efficacy, and the 2-methyl group in D region exhibited an agonist activity against TGR5. A significant breakthrough was achieved with the 3, 5-disubstituted aniline analog. Compound **13j** exhibited a potent FXR/TGR5 dual agonist activity.

Next, we investigated the effect of substitution pattern on aniline moiety (part D), as shown in Supplementary Fig. S1. A 3, 5-disubstituted pattern was important for FXR/TGR5 dual agonist activity. Compounds **13j** and **13k** showed dual potencies against both FXR and TGR5. However, **13m**, **13n**, and **13o** exhibited remarkably reduced activities against TGR5. The 2, 5-di- or 2, 3, 5-trisubstituted compounds **13p**, **13q**, and **13r** exhibited less potency in FXR agonist assay whereas they demonstrated good potency against TGR5. The 2-methyl-3-substituted compound **13s** showed a moderate FXR/TGR5 agonist activity. The 2-fluoro-3-substituted compounds **13t** and **13u** only showed strong FXR agonist activities, with a similar potency as OCA. These results suggested that the 3, 5-halogenated substitution pattern was essential for optimal FXR/TGR5 dual agonist potency.

We next sought to improve the dual potency against FXR/TGR5 by altering the substitution patterns on C ring (Supplementary Fig. S2.). Compounds **13v** and **13w** significantly decreased FXR potencies with an equipotent TGR5 agonist potency. In contrast, the 3-halogenated compounds, **13x** and **13y**, retained FXR potencies and reduced TGR5 potencies. The trifluoromethylpyridinyl group **13z** decreased the potencies against both FXR and TGR5. The 3-methoxy group of **13aa** showed a weak dual agonist activity. The pyridazine compound **13ab** did not have any activity against either FXR or TGR5. A lack of nitrogen in C ring of compounds **13ac**, **13ad**, **13ae**, **13af**, and **13ag** showed strong FXR activities. Compound **13ag** exhibited an FXR agonist activity of 136.5%, but it demonstrated a significantly low potency against TGR5. The results of Supplementary Figs. S1 and S2 suggest that a combination of substitutions at the C/D ring would create effective selective or dual agonist compounds against FXR and/or TGR5.

**Figure 3:**
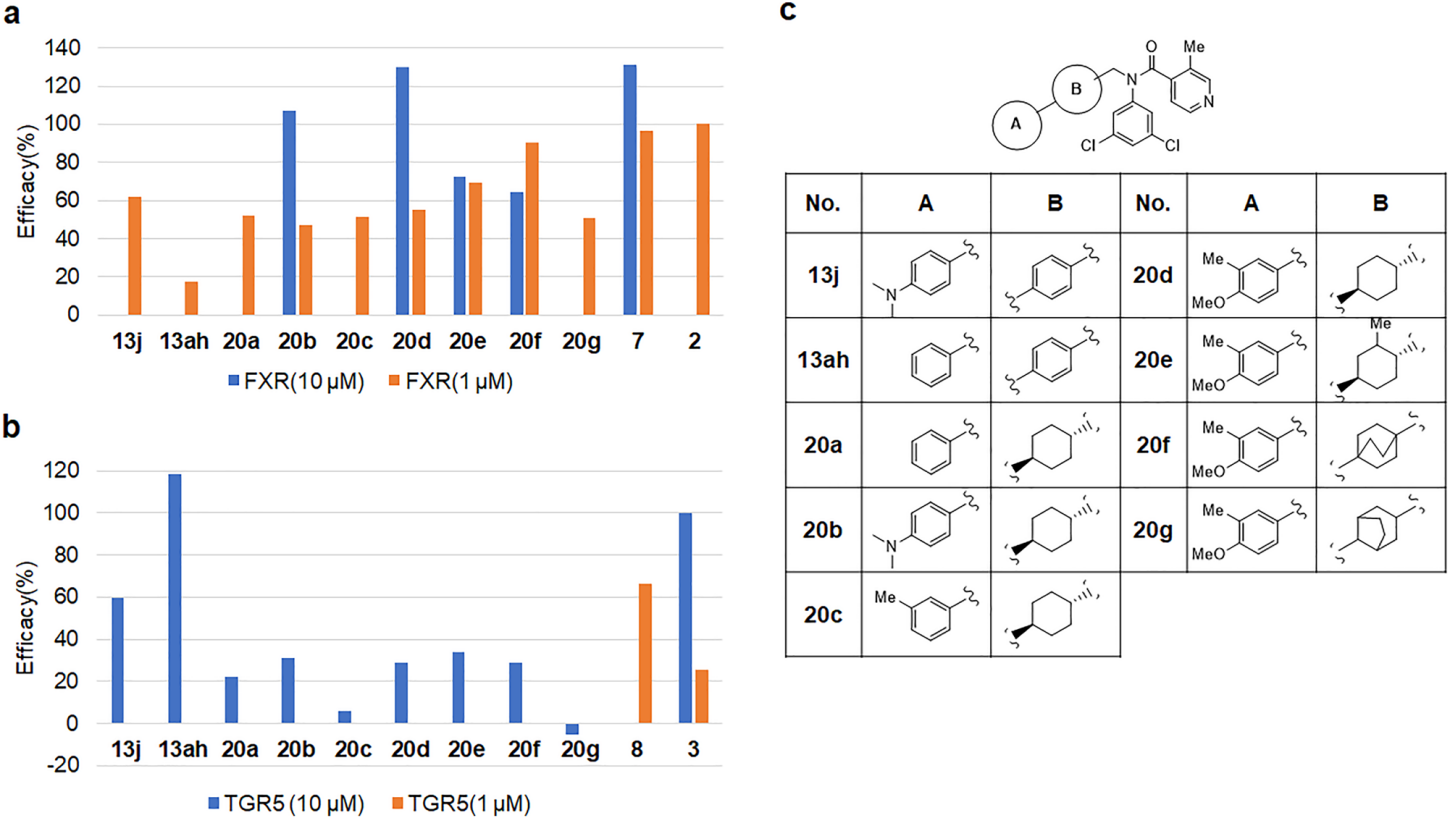
(**a**) FXR agonist activity of isonicotinamide derivatives **13j**, **13ah**, **20a**–**20g**. Efficacy(%); % vs OCA 1 µM as 100%, OCA; 100% (1 µM), 13.3% (0.1 µM); (**b**) TGR5 agonist activity of isonicotinamide derivatives **13j**, **13ah**, **20a**–**20g**. Efficacy(%); % vs INT-767 10 µM as 100%, INT-767 100% (10 µM), 25.5% (1 µM), 1.7% (0.1 µM). (**c**) Structures of isonicotinamide derivatives **13j**, **13ah**, **20a**–**20g**.

Some FXR agonist compounds with cyclohexyl or bicyclo groups in the B ring have been reported^39–47^. We synthesized cyclohexyl and bicyclo groups at the B ring, and we not only evaluated FXR agonist activity but also TGR5 agonist activity (Fig. 3). Although the des-dimethylamino group of **13ah** decreased the FXR agonist potency, its TGR5 agonist potency increased. Thus, compound **13ah** was a TGR5 full agonist (118.6% vs. INT-767). Compound **20a** exhibited a significantly increased FXR agonist activity but decreased TGR5 agonist activity. Similar trends were observed in **20d**, **20e**, and **20f**, which were more potent partial FXR agonists and weak TGR5 agonist. The bicyclo[2.2.2]octyl group fills the hydrophobic pocket in FXR LBD. We assumed that the bicyclic ring system would fill the central portion of binding pocket, and the bulkiness and tight conformation would cause a conformational resistance of Trp454. Merk et al. recently reported that a partial agonist DM175 (**9**) causes an outward movement of Trp454^48^. Compound **7** also moved Trp454 outward. LY2562175 (**6**) is also a partial agonist^49^.

**Figure 4:**
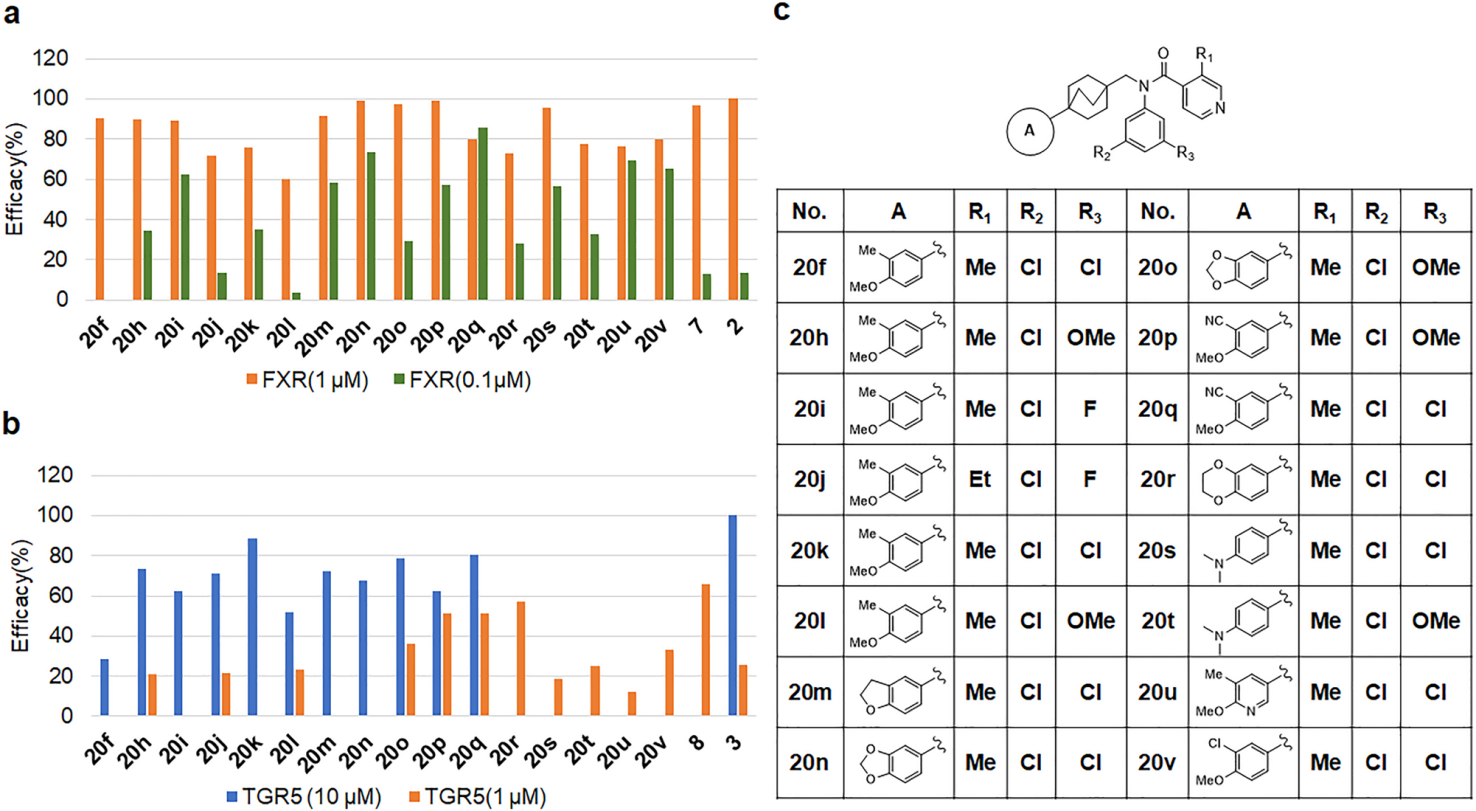
(**a**) FXR agonist activity of bicyclo[2.2.2]octane-isonicotinamide derivatives **20f**–**20v**. Efficacy(%); % vs OCA 1 µM as 100%, OCA; 100% (1 µM), 13.3% (0.1 µM); (**b**) TGR agonist activity of bicyclo[2.2.2]octane-isonicotinamide derivatives **20f**–**20v**. Efficacy(%); % vs INT-767 10 µM as 100%, INT-767 100% (10 µM), 25.5% (1 µM), 1.7% (0.1 µM). (**c**) Structures of bicyclo[2.2.2]octane-isonicotinamide derivatives **20f**-**20v**.

Finally, we targeted the substitution of part A because the substituent would interact with Trp454. Additional SAR investigations extensively explored the substitutions of aryl rings (A, C, and D rings, Fig. 4).

Compound **20f** increased the FXR agonist activity to a similar potency as OCA, although it decreased TGR5 agonist potency. To further explore the impact of a bicyclo B ring, we maintained the bicyclo ring in that region and concentrated our efforts on investigating substitutions around the ring. Compound **20h** showed a dual agonist activity. The 3, 4-dimethoxyphenyl group of compounds **20k** and **20l** exhibited decreased activities against FXR. Compounds **20m** and **20n** demonstrated improved potencies as FXR/TGR5 dual agonists. Compound **20o** exhibited a decreased FXR agonist potency, although TGR5 agonist potency was increased, as expected. Substitution of a nitrile moiety at the 3 position of benzene ring (A ring) in **20p** and **20q** provided a further improvement in FXR/TGR5 dual agonist potency. These compounds exhibited strong dual agonist potencies. Compound **20r** showed the most potent TGR5 activity, although its activity against FXR was less potent. Compound **20s** exhibited a high potency against FXR. Introduction of a nitrogen atom in A ring (**20u**) decreased the dual agonist potency. Replacement of the nitrile group with chloro group in compound **20v** decreased the agonist activity. Compounds **20p** and **20q** were promising candidates for FXR/TGR5 dual agonist.

Compound **7** is a potent FXR partial agonist in cell-based assays^31^. Compound **7** has been reported to possess a crystal structure of human FXR LBD^31^. The complex has a disordered 15-residue insertion region between helices 1 and 3. Compound **7** and its derivatives are partial agonists of FXR due to their decreased abilities to recruit coactivator SRC-1 as compared to that of the typical full agonist GW4064. The most notable impact of a partial agonist is the outward movement of Trp454 (Trp458 in PDB 1OSH) in compound **7** and the FXR-LBD complex^31^. The cocrystal structure indicated that this is the mechanistic difference between an agonist and partial agonist. A partial agonist causes an outward movement of Trp454 and destabilizes a loop connecting helix 12. In the agonistic conformation, the ligand interacts with Trp454 and His447, and His447 makes an edge-to-face stacking interaction with Trp469 in helix 12 (AF2), thus, stabilizing it.

Merk et al. recently reported the partial agonist DM175 and its cocrystal structure with FXR LBD^48^. Binding of DM175 causes an outward movement of Trp454, which is driven by site occupation by the tert-butyl moiety. The binding mode of DM175 is obviously different from the structure of the complex containing full agonists such as OCA, CDCA, and GW4064.

**Figure 5:**
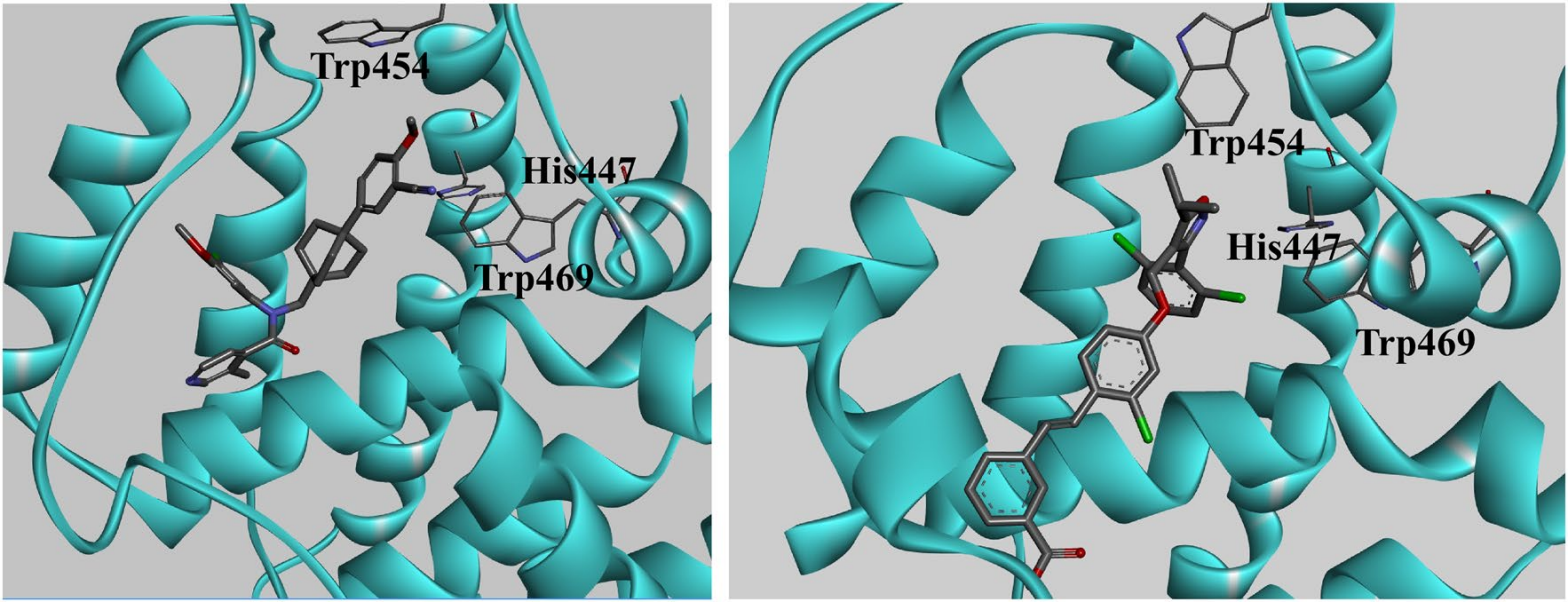
(**a**) Model showing the docking of compound **20p** into the FXR ligand binding domain obtained from the X-ray crystal structure PDB 1OSH. (**b**) GW4064 (**4**) is shown for comparison (3DCT).

The agonist mechanism of action for FXR LBD is through interaction with Trp469 and His447. Moreover, the interaction with Trp454 plays an important role between FXR LBD and agonist ligands. We examined the computational molecular docking of **20p** into the X-ray structure of FXR LBD (PDB 1OSH, Fig. 5a). The cocrystal structure of GW4064 with the active site of FXR protein has been reported (Fig. 5b)^30^. The isoxazole moiety of GW4064 interacts with Trp454 and His447 in helix 11, and His447 forms an edge-to-face stacking interaction with Trp469 located in helix 12 (AF2), which stabilizes Trp458 and recruits the co-activator. Compound **7** causes an outward movement of Trp458, thus, inducing a partial agonist activity against FXR. Ivermectin, a widely used antiparasitic drug with a distinct chemical structure from GW4064 and compound **7**, promoted coactivator recruitment by FXR. Ivermectin enhanced the interaction of FXR with various coactivator LXXLL motifs from the family of steroid receptor coactivators such as SRC1, SRC2, and SRC3. Since its interaction is less than that of GW4064, ivermectin is a partial agonist^50^. However, ivermectin also induces the recruitment of corepressor NCoR-2 by FXR, which is not exhibited by GW4064. The results indicate a partial agonist activity of ivermectin with unique properties that modulate coregulator recruitment. The cocrystal structure of ivermectin with a corepressor instead of a coactivator showed a disordered helix 12 and loop between helices 11 and 12. Additionally, helix 12 is shortened such that the structure contains no activator but a corepressor. Therefore, the cocrystal structure between ivermectin and FXR-LBD is the antagonist form.

**Figure 6:**
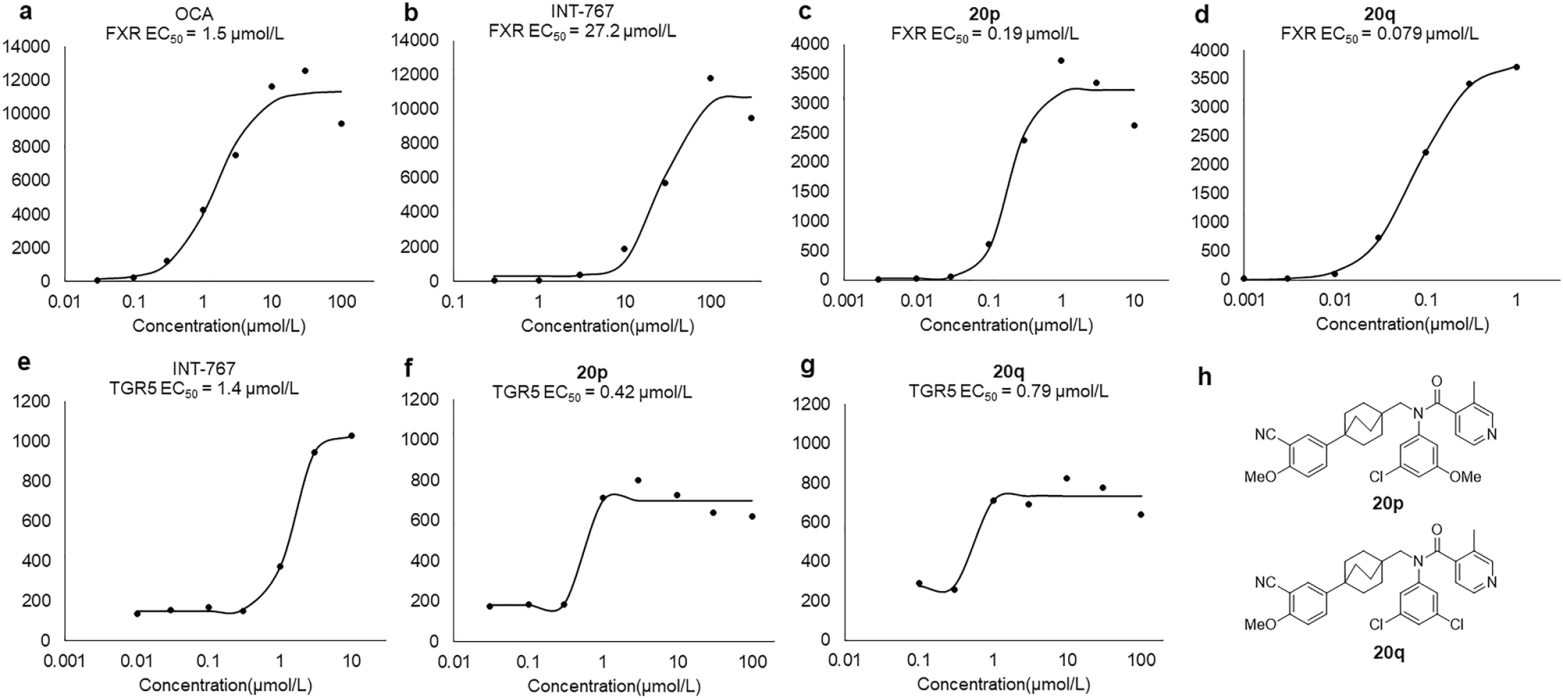
Concentration–response curves. **(a)** OCA FXR EC_50_ value; (**b**) INT-767 FXR EC_50_ value; (**c**) **20p** FXR EC_50_ value; (**d**) **20q** FXR EC_50_ value; (**e**) INT-767 TGR5 EC_50_ value; (**f**) **20p** TGR5 EC_50_ value; (**g**) **20q** TGR5 EC value; (**h**) Structures of **20p** and **20q**.

The compounds **20p** and **20q** were used to measure the EC_50_ value. The potencies of **20p** (FXR EC_50_ = 190 nM, Emax = 28%, TGR5 EC_50_ = 420 nM) and **20q** (FXR; EC_50_ = 79 nM, Emax = 33%, TGR5; EC_50_ = 790 nM) were stronger than those of OCA and INT-767 (Fig. 6).

Pharmacokinetic profiles of the bicyclo compounds **20p** and **20q** were evaluated in rats (Supplementary Table S1). The in vivo pharmacokinetic study revealed that **20p** and **20q** exhibited a rather low plasma exposure at an oral dose of 3 mg/kg with *C*max values of 50.3 and 3.20 ng/mL and *T*_1/2_ values of 1.45 and 4.52 h, respectively. These results suggested that an intestinal exposure to **20p** and **20q** after oral dosing may be sufficient to activate TGR5 in the intestine, increase GLP-1 secretion, and decrease blood glucose levels. Moreover, Fang et al. recently reported that compound **7** acts against intestinal FXR and activates FXR agonist potency without being absorbed^51^. Therefore, compounds **20p** and **20q** were selected for further evaluation in C57BL/6J mice, that were administered a choline-deficient amino acid-defined high-fat diet (CDAHFD) consisting 60 kcal% fat and 0.1% methionine by weight for 1 week^52^. This model was reported an elevated ALT level, which was a parameter to detect liver injury. Histopathological results indicated hepatocellular steatosis after 1 week. CDAHFD was fed for 6 weeks, and fibrosis developed in C57BL/6J mice.

**Figure 7:**
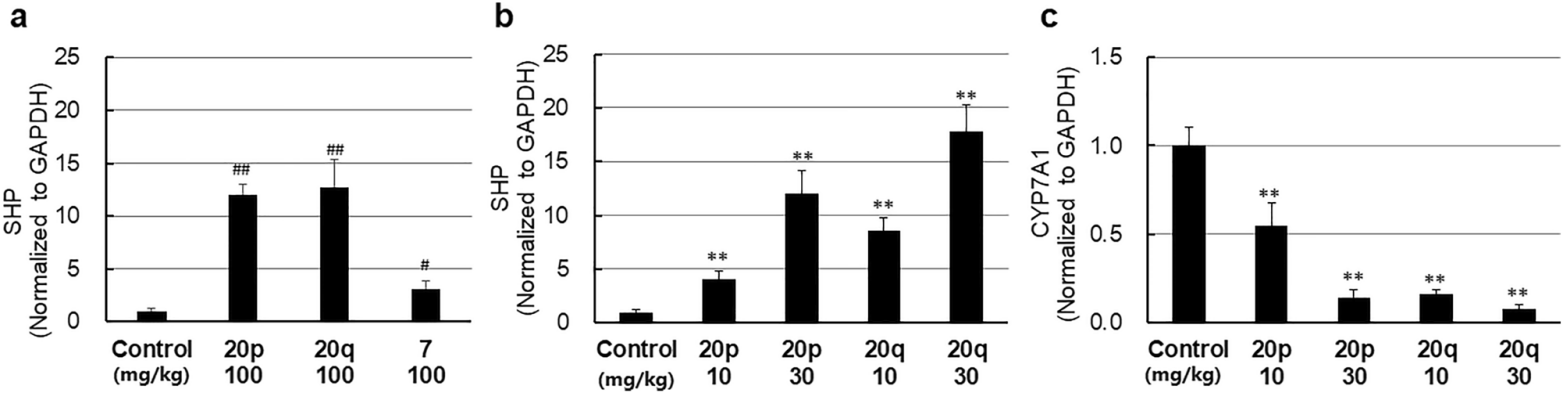
(**a**) Induction of SHP mRNA in mice liver by **20p** and **20q** following a single oral dose (100 mg/kg), compared with fexaramine **7** (100 mg/kg). (**b**) Induction of SHP mRNA in mice liver by **20p** and **20q** (10, 30 mg/kg). ^#^p < 0.05, ^##^p < 0.01, significant difference from a control group, as analyzed by Student’s t-test., **p < 0.01 significant difference from a control group, as analyzed by Dunnett’s multiple comparison test. n = 5, control n = 10. (**c**) Repression of CYP7A1 in mice liver by **20p** and **20q** following a single oral dose (10, 30 mg/kg). **p < 0.01 significant difference from the control group, as analyzed by Dunnett’s multiple comparison test. n = 5, control n = 10.

The FXR target genes, SHP mRNA and CYP7A1, were measured using real-time PCR after a single dose of **20p** and **20q** (100, 30, and 10 mg/kg). Compound **7** is an FXR agonist, and it increases SHP mRNA in the liver of mice^53^. Both compounds, **20p** and **20q**, dose-dependently induced SHP mRNA in the liver at 100, 30, and 10 mg/kg (Fig. 7a,b) and decreased CYP7A1 in the liver at 30 and 10 mg/kg (Fig. 7c). These results indicated that **20p** and **20q** are in vivo FXR agonists.

**Figure 8:**
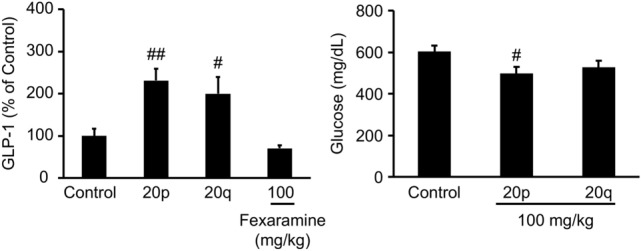
(**a**) GLP-1 secretion study of **20p** and **20q** in C57 mice. (**b**) Blood glucose levels of **20p** and **20q** on OGTT in C57 mice. ^#^p < 0.05, ^##^p < 0.01, significant difference from the control group, as analyzed by Student’s t-test.

After identifying the compounds with a high potency against TGR5, **20p** and **20q** were evaluated for in vivo GLP-1 secretion in C57BL/6J mice. A single oral administration of **20p** and **20q** at a dose of 100 mg/kg increased plasma GLP-1 levels, as assessed by an oral glucose tolerance test (OGTT). **20p** and **20q** significantly increased the plasma levels of GLP-1 (Fig. 8a).

Compounds **20p** and **20q** elevated the plasma levels of GLP-1, and a high concentration of GLP-1 could lower blood glucose levels. The favorable GLP-1 secretion results encouraged us to further evaluate the in vivo efficacy in C57 mice. A single oral dose of **20p** robustly lowered the blood glucose level, although a single oral dose of **20q** did not reduce the blood glucose level (Fig. 8b).

Compound **20p** was screened against a broad panel of NRs and GPCRs to determine its agonist activity in vitro. Compound **20p** showed no significant off-target activity against GPCRs and NRs. Binding assays for RXR alpha, ER alpha, and ER beta (EC_50_ > 30 µM) and cellular and NR functional assays for GPR40, GPR120, GPR119, LXR alpha, LXR beta, CAR, PPAR alpha, PPAR delta, PPAR gamma, and PXR (EC_50_ > 30 µM) are included in additional data of S4 Supplementary information.

## Discussion

We identified **20p** (FXR EC_50_ = 190 nM, Emax = 28%, TGR5 EC_50_ = 420 nM) as a non-bile acid FXR/TGR5 dual agonist. The potency of **20p** was stronger than those of OCA and INT-767 in vitro assay. Compound **20p** was evaluated in C57BL/6 J mice, that were administered CDAHFD consisting of 60 kcal% fat and 0.1% methionine by weight for one week. Elevated ALT levels were observed in the model after one week of initiation of feeding, and histopathological results indicated hepatocellular steatosis after 1 week^52^. Moreover, after feeding CDAHFD for 6 weeks, fibrosis was developed in C57BL/6J mice^52^. Compound **20p** dose-dependently induced SHP mRNA and decreased CYP7A1 expression in the liver. The compound **20p** significantly increased the GLP-1 level in C57BL/6J mice. The in vivo glucose-lowing study indicated that a single oral dose of **20p** significantly reduced blood glucose levels. These results indicate that **20p** is an FXR/TGR5 dual agonist.

Although several potent steroidal FXR/TGR5 dual agonists have been reported, no non-steroidal FXR/TGR5 dual agonist has been reported to date. A variety of isonicotinamide derivatives were designed, synthesized, and evaluated as a series of highly potent non-bile acid FXR/TGR5 dual agonists. The EC_50_ values of agonist activities against FXR and TGR5 for some isonicotinamide derivatives were below 1 µM. An in vivo glucose-lowering study reported that a single oral dose of **20p** significantly reduces blood glucose levels. Compound **20p** significantly increased the GLP-1 level. Intestine-restricted FXR agonist compound **7** is known to stimulate serum GLP-1 secretion in wild-type C57BL/6J mice for 7 days but not in TGR5^-/-^ or FXR^-/-^ mice^54^. In our model, a single dose of compound **7** did not increase serum GLP-1 in the wild-type mice. It has been reported that compound **7** modulates gut microbiomes and turns taurochenodeoxycholic acid into lithocholic acid (LCA) by the gut microbiomes, and the LCA stimulates TGR5 expression and TGR5-mediated cAMP signaling and increases GLP-1 secretion^54^. The GLP-1 secretion of compounds **20p** and **20q** would be a direct action of TGR5 agonist activities via TGR5.

## Methods

### In vitro FXR agonist assay protocol

To directly evaluate the in vitro agonist activity towards FXR, test compounds were evaluated in CHO cells transfected with FXR responsive luciferase reporter (Nuclear Receptor & In Vitro Toxicology Solutions, Indigo Biosciences, State College, PA, USA). Cells were incubated with the test compounds for 22 h, and potency was assessed using Multi-Mode Microplate Reader (FlexStation 3, Molecular Devices Inc., San Jose, CA, USA). Efficacies are reported relative to OCA, which was set as 100% FXR activation at a concentration of 1 µM. Each compound was tested in duplicate, and the average value was reported^35,36^.

The cell recovery medium (CRM, Human Farnesoid X Receptor (NR1H4, FXR reporter assay system, Indigo Biosciences, State College, PA, USA) and compound screening medium (CSM, Human Farnesoid X Receptor (NR1H4, FXR reporter assay system, Indigo Biosciences, State College, PA, USA) were removed from the freezer and thawed in a water bath at 37 °C. Test compounds were dissolved in dimethyl sulfoxide (DMSO, FUJIFILM Wako Pure Chemical Corporation, Osaka city, Osaka, Japan), and the treatment medium was prepared and diluted with CSM to achieve a final concentration of 0.2% total DMSO. The reporter cells were thawed by transferring 3.3 mL CRM at 37 °C into tubes of frozen cells. The tube containing reporter cells was recapped, and it was immediately place in a 37 °C water bath for 5 min. The reporter cells were gently inverted several times, and 100 µL cell suspension was dispensed into each well. One hundred microliters of treatment media was added into wells. Subsequently, the assay plate was kept at 37 °C, and it was incubated with 5% CO_2_ for 22 h. To prepare luciferase detection reagent (LDR), the detection substrate was gently mixed with detection buffer, media contents were removed from each well, and 100 µL LDR was added to each well of the assay plate. The assay plate was allowed to rest at room temperature for 5 min, and luminescence was quantified using the Multi-Mode Microplate Reader (FlexStation 3, Molecular Devices Inc., San Jose, CA, USA)^36^.

### In vitro TGR5 agonist assay protocol

TGR5 agonist activity was evaluated by transfecting CHO cells with TGR5 using the Discover X expression kit (cAMP Hunter eXpress Assay Kit, Discover X Fremont, CA, USA). Cells were incubated with the test compounds for 0.5 h, and intracellular cAMP levels were measured using Multi-Mode Microplate Reader (FlexStation 3, Molecular Devices Inc., San Jose, CA, USA). The response to 10 µM INT-767 **3** was set as 100% TGR5 activation^37^.

Each compound was tested at concentrations of 10, 1, or 0.1 µM in duplicate (n = 2), with the average value shown. EC_50_ value of compounds **20p** and **20q** were determined by using a 4 parameter logistic equation, *Y* = min + (max − min)/(1 + 10^(n * (log10(EC_50_) − log10(conc)))), in nonlinear least squares fitting using the Microsoft Excel Solver^55^.

### In vivo assay protocol (FXR)

Test compounds were orally administered to 7-week old male C57BL/6J mice (Nippon Charles River Co., Ltd., Yokohama City, Kanagawa, Japan), that were administered CDAHFD (#A06071302, Research Diets, 20 Jules Lane, New Brunswick, NJ, USA) consisting 60 kcal% fat and 0.1% methionine by weight for 7 days. After four hours of administration, the liver was removed, and total RNA was extracted using a Pure Link RNA Mini Kit (Invitrogen, Thermo Fisher Scientific K. K., Minato-ku, Tokyo, Japan). The concentration of total RNA was measured using Micro Sample Spectrophotometer (SimpliNano, GE Healthcare UK Ltd, Amersham Place Little Chalfont Buckinghamshire, HP7 9NA UK), and cDNA was synthesized using ReverTra Ace qPCR RT Master Mix (Toyobo Co., Ltd., Osaka City, Osaka, Japan). Real-time PCR was performed using a CFX96 Touch Real-Time PCR Analysis System (CFX Manager, Bio-Rad, 1000 Alfred Nobel Drive, Hercules, CA, USA), and the mRNA expression levels of SHP and CYP7A1, the target genes of FXR, were measured using the intercalator method^52^.

### In vivo assay protocol (GLP-1)

Measurement of GLP-1 secretion: To measure plasma GLP-1 levels, 100 mg/kg of compounds **20p** and **20q** were administered orally to overnight-fasted C57 mice (n = 5 animals/group). One hour later, all mice were challenged with 2 g/kg glucose, and blood samples were collected after 15 min of glucose challenge test. Plasma GLP-1 levels were measured using the GLP-1 ELISA Kit Wako, High Sensitive (FUJIFILM Wako Pure Chemical Corporation, Osaka city, Osaka, Japan)^56^.

### In vivo assay protocol (OGTT test)

To measure blood glucose levels, 100 mg/kg of test compounds **20p** and **20q** or vehicle were administered orally to overnight-fasted C57 mice (n = 5 mice/group, control; n = 10). One hour later, all mice were challenged with 2 g/kg glucose, and blood samples were collected after 15 min of glucose loading. The blood glucose levels were measured using LabAssay Glucose (FUJIFILM Wako Pure Chemical Corporation, Osaka City, Osaka, Japan)^56^.

### In vivo in-house animal guidelines

Study protocols were designed and refined by taking reduction of animal use into consideration. The study protocol was approved by the Animal Care and Utilization Committee of Fuji Yakuhin Research Laboratories, and all methods complied. We carried out in the compliance with ARRIVE guidelines. A statement to confirm that all methods were carried out in accordance with relevant guidelines and regulations.

### General synthesis

General synthesis procedures were conducted as described in Supplementary S5.

The general synthesis of isonicotinamide derivative **13** was conducted as described in Supplementary Fig. S3.

Substituted-phenyl-cycloalkyl compounds were synthesized as described in Supplementary Fig. S4^40^.

Substituted-phenyl-bicyclo[2,2,2]octane analogs were synthesized as described in Supplementary Fig. S5 (route 1)^43^. Some substituted-phenyl-bicyclo[2,2,2]octane compounds were synthesized^43–47^.

The direct cross-coupling of substituted aromatic ring (A region) and bicyclo[2,2,2]octane (B region) is effective for the synthesis and evaluation of bicyclo[2,2,2]octane compounds. Baran et al. recently reported a cross-coupling method of Redox-Active Ester (RAE) with organometallic species such as a borane, nickel, and iron-catalyst^57–61^. Using an iron-based organometallic catalyst, such as tris(2,4-pentanedionato)iron (III) (Fe(acac)_3_), enables the formation of the C–C bond between tertiary alkyl carboxylic acids and aryl magnesium coupling^59,61^. The iron-catalyzed cross-coupling of RAE was a good opportunity for us to optimize the A ring region with bicyclo[2,2,2]octane derivatives. We synthesized C–C coupling of the *N*-hydroxyphthalimide-based ester compound **35** derived from 4-(methoxycarbonyl) bicyclo[2,2,2]octane-1-carboxylic acid **31** with Fe(acac)_3_ and aryl magnesium bromide **36** in the presence of 1,3-dimethyl-3,4,5,6-tetrahydro-2(1*H*)-pyrimidinone, as shown in Supplementary Fig. S6.

## Supplementary Information

Supplementary Information.
