# Supplementary material for: Discovery, optimization, and evaluation of non-bile acid FXR/TGR5 dual agonists
Source: Sci Rep. 2021 Apr 28;11:9196. doi: 10.1038/s41598-021-88493-0 (PMC8080777; doi:10.1038/s41598-021-88493-0)
Supplement: Supplementary file 1 — Supplementary Information. [file 41598_2021_88493_MOESM1_ESM.pdf]

Discovery, Optimization, and Evaluation of Non-Bile Acid FXR/TGR5 Dual Agonists

Sachiho Miyata[1], Yuji Kawashima[1], Miku Sakai[2], Masaya Matsubayashi[2], Keisuke Motoki[2], Yui Miyajima[1], Yousuke Watanabe[2], Noriko Chikamatsu[2], Tetsuya Taniguchi[2] and Ryukou Tokuyama[1,*].

[1]Research Laboratory 1, FUJI YAKUHIN. CO., LTD. 1-32-3, Nishi-Omiya, Nishi-ku, Saitama City, Saitama, Japan. [2]Research Laboratory 2, FUJI YAKUHIN. CO., LTD. 636-1, Iida-Shinden, Nishi-ku, Saitama City, Saitama, Japan.

Complimentary Information

Contents

S1. Supplementary Figure S1. Structure activity relationships of 4-dimethylamino biphenylisonicotinamide derivatives.

S2. Supplementary Figure S2. Structure activity relationships of 4-4-dimethylaminobiphenyl derivatives derivatives.

S3. Supplementary Table S1. Pharmacokinetic properties of **20p** and **20q** after oral administration to rats.

S4. In vitro, in vivo assay and selectvity.

S5. General synthesis procedures.

S6. Supplementary Figure S3. General synthesis of *N*-biarylmethyl-*N*-(3,5-dichlorophenyl)-3-methylisonicotinamide derivatives **13**

S7. Supplementary Figure S4. Synthesis of *N*-substituted-phenyl-cycloalkyl-(3,5-dichlorophenyl)-3-methylisonicotinamide **20**.

S8. Supplementary Figure S5. Synthesis of *N*-substituted-aryl bicyclo[2,2,2]-octan-1-yl-methyl-(3,5-dichlorophenyl)-3-methylisonicotinamide **20** (route 1).

S9. Supplementary Figure S6. Synthesis of *N*-4-substituted-aryl bicyclo[2,2,2]-octan-1-yl-methyl-(3,5-dichlorophenyl)-3-methylisonicotinamide **20** (route 2).

S10. Preparation of **13j**

S11. NMR and LCMS data of **13e-g**, **13k-l**, **13p-r**, **13v-ah**

S12. Preparation of **13m**

S13. NMR and LCMS data of **13a-d**, **13h-i**, **13u**

S14. Preparation of **13n**

S15. Preparation of **13o**

S16. NMR and LCMS data of **13s-t**

S17. Preparation of **20d**

S18. NMR and LCMS data of **20a-c, 20e**

S19. Preparation of **20p** (route 1)

S20. NMR and LCMS data of **20f, 20h-j**, **20q**.

S21. Preparation of **30r** (route 2)

S22. NMR and LCMS data of **20k-o**, **20r-v**

S23. Preparation of **20g**

S24. Supplementary Table S8. Structure activity relationships of 4-dimethylamino biphenyl derivatives.

S25. Supplementary Table S9. Structure activity relationships of 4-dimethylamino biphenylisonicotinamide derivatives.

S26. Supplementary Table S10. Structure activity relationships of 4-4-dimethylaminobiphenyl derivatives derivatives.

S27. Supplementary Table S11. Structure activity relationships of isonicotinamide derivatives .

S28. Supplementary Table S12. Structure activity relationships of bicyclo[2.2.2]octane-isonicotinamide derivatives.

S29. Docking and computational method.

S30. Reference

S1. Supplementary Figure S1. Structure activity relationships of 4-dimethylamino biphenylisonicotinamide derivatives.

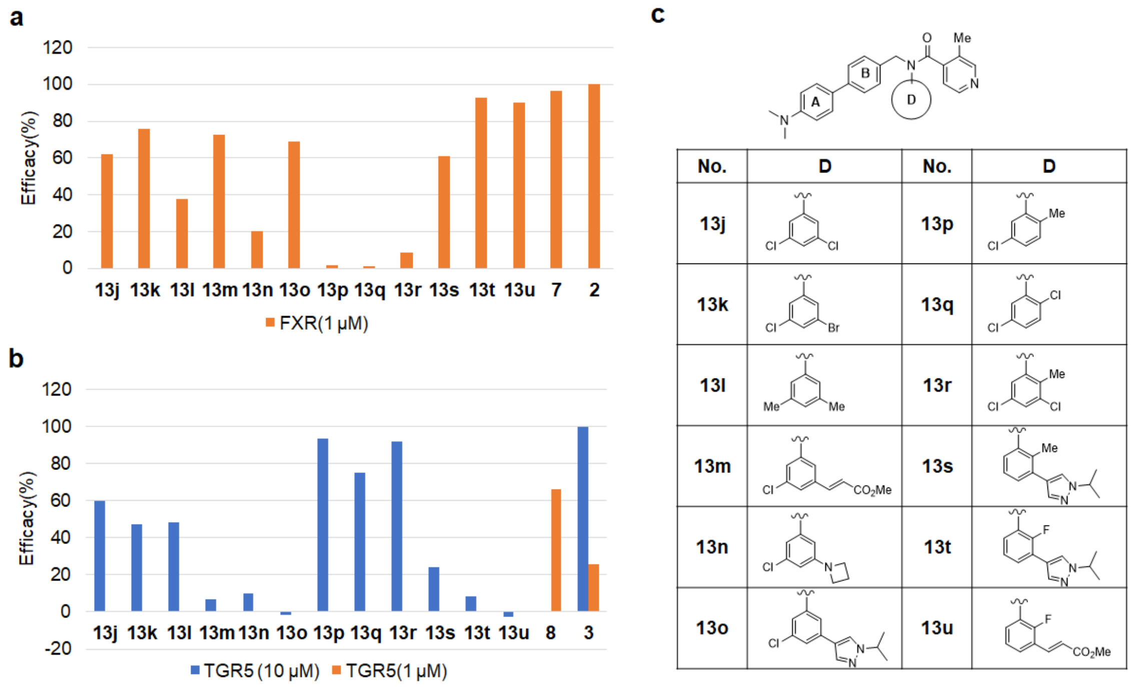

Figure S1. (**a**) FXR agonist acitivity of 4-dimethylamino biphenylisonicotinamide derivatives **13j-13u**. Efficacy(%); % vs OCA 1 μM as 100%, OCA; 100% (1 μM), 13.3% (0.1 μM); (**b**) TGR5 agonist activity of 4-dimethylamino biphenyl derivatives **13j-13u**. Efficacy(%); % vs INT-767 10 μM as 100%, INT-767 100% (10 μM), 25.5% (1 μM), 1.7% (0.1 μM). (**c**) Structures of 4-dimethylamino biphenyl derivatives **13j-13u**.

S2. Supplementary Figure S2. Structure activity relationships of 4-dimethylaminobiphenyl derivatives derivatives.

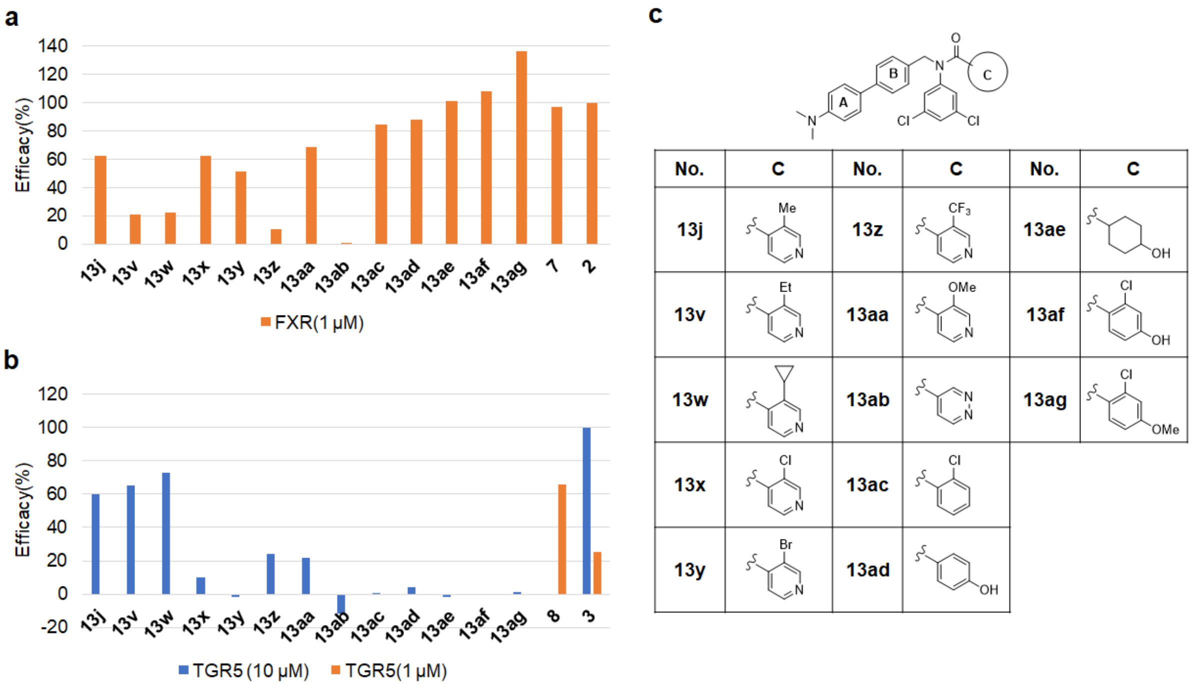

Figure S2. (**a**) FXR agonist acitivity of 4-dimethylamino biphenyl derivatives **13j**, **13v-13ag**. Efficacy(%); % vs OCA 1 μM as 100%, OCA; 100% (1 μM), 13.3% (0.1 μM); (**b**) TGR5 agonist activity of 4-dimethylamino biphenyl derivatives **13j**, **13v-13ag**. Efficacy(%); % vs INT-767 10 μM as 100%, INT-767 100% (10 μM), 25.5% (1 μM), 1.7% (0.1 μM). (**c**) Structures of 4-dimethylamino biphenyl derivatives **13j**, **13v-13ag**.

S3. Supplementary Table S1. Pharmacokinetic properties of **20p** and **20q** after oral administration to rats. [a]

Determination of Drug Pharmacokinetics in Sprague-Dawley rats.

Blood samples (about 200 μl) were obtained from the jugular vein at 0.083 (intravenous only), 0.25, 0.5, 1, 2, 4, 8, and 24 hours from 16-hour fasted rats. Plasma was obtained from the blood samples by centrifugation at 3000 rpm for 10 minutes at 4 °C. Plasma were deproteinated and centrifuged at 3000 rpm for 10 minutes at 4 °C. Drug concentrations were measured using LC-MSMS (Triple Quad 4500; Waters Corporation, Milford, MA). Area under the plasma concentration-time curve at 0–24h (AUC0–24h) were calculated using the trapezoidal rule. pharmacokinetic parameters were calculated using the Phoenix WinNonlin 6.4 software (Certara L.P. Princeton, NJ).

Compound	$T_{1/2}$ (hr)	Cmax (ng/mL)	AUC (ng hr/mL)	CLtot (mL/hr/kg)
**20p** (p.o)	1.45	50.3	120.64	25616
**20q** (p.o)	4.52	3.20	10.97	159140
**20q** (i.v)	5.04	2584	1462	2050

[a] n=3, 3mg/kg

S4. *In vitro*, *in vivo* assay, and selectvity.

To directly evaluate the *in vitro* agonist activity towards FXR, test compounds were evaluated in CHO cells transfected with FXR responsive luciferase reporter (Nuclear Receptor & *In Vitro* Toxicology Solutions, Indigo Biosciences, State College, PA, USA). Cells were incubated with the test compounds for 22 h, and potency was assessed using Multi-Mode Microplate Reader (FlexStation 3, Molecular Devices Inc., San Jose, CA, USA). Efficacies are reported relative to OCA, which was set as 100% FXR activation at a concentrati with FXR responsive luciferase reporter on of 1 μM. Each compound was tested in duplicate, and the average value was reported.[1]

The cell recovery medium (CRM, Human Farnesoid X Receptor (NR1H4, FXR reporter assay system, Indigo Biosciences, State College, PA, USA) and compound screening medium (CSM, Human Farnesoid X Receptor (NR1H4, FXR reporter assay system, Indigo Biosciences, State College, PA, USA) were removed from the freezer and thawed in a water bath at 37 °C. Test compounds were dissolved in dimethyl sulfoxide (DMSO, FUJIFILM Wako Pure Chemical Corporation, Osaka city, Osaka, Japan), and the treatment medium was prepared and diluted with CSM to achieve a final concentration of 0.2% total DMSO. The reporter cells were thawed by transferring 3.3 mL CRM at 37 °C into tubes of frozen cells. The tube containing reporter cells was recapped, and it was immediately place in a 37°C water bath for 5 min. The reporter cells were gently inverted several times, and 100 μL cell suspension was dispensed into each well. One hundred microliters of treatment media was added into wells. Subsequently, the assay plate was kept at 37 °C, and it was incubated with 5% CO_2 for 22 h. To prepare luciferase detection reagent (LDR), the detection substrate was gently mixed with detection buffer, media contents were removed from each well, and 100 μL LDR was added to each well of the assay plate. The assay plate was allowed to rest at room temperature for 5 min, and luminescence was quantified using the Multi-Mode Microplate Reader (FlexStation 3, Molecular Devices Inc., San Jose, CA, USA).[1]

***In vitro* TGR5 agonist assay protocol**

TGR5 agonist activity was evaluated by transfecting CHO cells with TGR5 using the Discover X expression kit (cAMP Hunter eXpress Assay Kit, Discover X Fremont, CA, USA). Cells were incubated with the test compounds for 0.5 h, and intracellular cAMP levels were measured using Multi-Mode Microplate Reader (FlexStation 3, Molecular Devices Inc., San Jose, CA, USA). The response to 10 μM INT-767 **3** was set as 100% TGR5 activation.[2]

Each compound was tested at concentrations of 10, 1, or 0.1 μM in duplicate (n=2), with the average value shown. EC_{50} value of compounds **20p** and **20q** were determined by using a 4 parameter logistic equation, Y = min + (max - min)/(1 + 10^(n * (log10(EC_{50}) - log10(conc)))), in nonlinear least squares fitting using the Microsoft Excel Solver.[3]

***In vivo* assay protocol (FXR)**

Test compounds were orally administered to 7-week old male C57BL/6J mice (Nippon Charles River Co., Ltd., Yokohama City, Kanagawa, Japan), that were administered CDAHFD (#A06071302, Research Diets, 20 Jules Lane, New Brunswick, NJ, USA) consisting 60 kcal% fat and 0.1% methionine by weight for 7 days. After four hours of administration, the liver was removed, and total RNA was extracted using a Pure Link RNA Mini Kit (Invitrogen, Thermo Fisher Scientific K. K., Minato-ku, Tokyo, Japan). The concentration of total RNA was measured using Micro Sample Spectrophotometer (SimpliNano, GE Healthcare UK Ltd,

Amersham Place Little Chalfont Buckinghamshire, HP7 9NA UK), and cDNA was synthesized using ReverTra Ace qPCR RT Master Mix (Toyobo Co., Ltd., Osaka City, Osaka, Japan). Real-time PCR was performed using a CFX96 Touch Real-Time PCR Analysis System (CFX Manager, Bio-Rad, 1000 Alfred Nobel Drive, Hercules, CA, USA), and the mRNA expression levels of SHP and CYP7A1, the target genes of FXR, were measured using the intercalator method.[4]

***In vivo* assay protocol (GLP-1)**

Measurement of GLP-1 secretion: To measure plasma GLP-1 levels, 100 mg/kg of compounds **20p** and **20q** were administered orally to overnight-fasted C57 mice (n=5 animals/group). One hour later, all mice were challenged with 2 g/kg glucose, and blood samples were collected after 15 min of glucose challenge test. Plasma GLP-1 levels were measured using the GLP-1 ELISA Kit Wako, High Sensitive (FUJIFILM Wako Pure Chemical Corporation, Osaka city, Osaka, Japan).[5]

***In vivo* assay protocol (OGTT test)**

To measure blood glucose levels, 100 mg/kg of test compounds **20p** and **20q** or vehicle were administered orally to overnight-fasted C57 mice (n=5 mice/group, control; n=10). One hour later, all mice were challenged with 2 g/kg glucose, and blood samples were collected after 15 min of glucose loading. The blood glucose levels were measured using LabAssay Glucose (FUJIFILM Wako Pure Chemical Corporation, Osaka City, Osaka, Japan).[5]

***In vivo* in-house animal guidelines**

Study protocols were designed and refined by taking reduction of animal use into consideration. The study protocol was approved by the Animal Care and Utilization Committee of Fuji Yakuhin Research Laboratories, and all methods complied. We carried out in the compliance with ARRIVE guidelines. A statement to confirm that all methods were carried out in accordance with relevant guidelines and regulations.

Selectivity

The selectivity tests of compound **20p** were investigated by Eurofine Cerep (86600, Celle-Levescault, France) and Eurofine Panlabs Discovery Services (No. 25, Wugong, 6th Road, Wugu District, New Taipai City, Taiwan). Compound binding was caluculated as a % inhibition of the binding of radioactively labeled ligand specific for each target. Cellular agonist effect wa calculated as a % of control response to a known reference agonist for each target and cellular antagonist effect as caluculated as a % inhibition of control reference agonist response for each target at 30 μM of compound **20p**.

GPR40 5.9% ; GPR120 -5.2%; GPR119 1.8%; LXR alpha 0.2%; LXR beta 3.7%, RXR alpha -12.3%; CAR -2.5%, PPAR alpha 0.0%; PPAR delta -3.6%; PPAR gamma -1.0%; PXR 4.5%; ER alpha -16.6%; ER beta 17% at 30 μM.

S5. General synthesis procedure.

All solvents and chemicals were used as purchased without further purification. The progress of all reactions was monitored on Merck precoated silica gel plates (with the fluorescence indicator UV254, Merck KGaA, 64271, Darmstadt, Germany) using ethyl acetate (AcOEt) /*n*-hexane as the solvent system. Column chromatography was performed with silica gel 60 (200-400 mesh) with the solvent mixture specified. Spots were visualized by irradiation with ultraviolet light (254 nm) or by staining with phosphomolybdic acid and warming. NMR spectra were recorded on a JEOL JNM-ECZ400S/L1 spectrometer and internally referenced to tetramethylsilane (TMS). Chemical shifts are reported in parts per million (ppm). The following abbreviations are used: br = broad signal, s = singlet, d = doublet, dd = doublet of doublets, t = triplet, q = quartet, and m = multiplet. ^{13}C-NMR spectra were recorded on a JEOL JNM-ECZ400S/L1 spectrometer (100.5MHz) and internally referenced to solvent peak. Chemical shifts are reported in parts per million (ppm). Peaks assigned to common solvents such as DMSO-d_6. Ultra-performance liquid chromatography (UPLC) and mass spectrometry on Waters ACQUITY UPLC H-Class/SQD2 and MassLynx workstation system were employed to determine the purity of the tested compounds. The purity of all final compounds was ≥ 95%. An LC/MS instrument with a Gemini C18 (ODS) 1.7 µm (2.1 x 50 mm) column and mobile phases A: 0.1% formic acid (FA) in H_2O pH 2.7 and B: 0.1% FA in acetonitrile (CH_3CN) were used in Method A with the following gradient mode: from 30 to 100% B in 5 min with a gradient. UV DAD detection occurred at λ = 254 nm. For Method B, a 1.7 µm (2.1 x 30 mm) column and mobile phase A: 0.1% FA in H_2O pH 2.7 and mobile phase B: 0.1% FA CH_3CN were used in gradient mode: from 30 to 100% B in 3 min with a gradient. UV DAD detection was performed at λ = 254 nm.

S6. Supplementary Figure S3. General synthesis of *N*-biarylmethyl-*N*-(3,5-dichlorophenyl)-3-methylisonicotinamide derivatives **13**.[a]

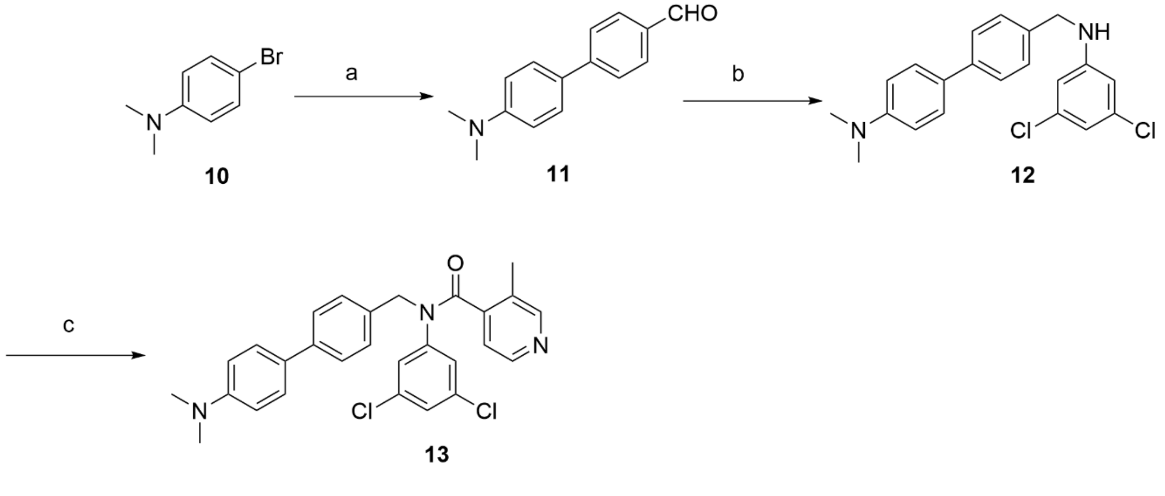

[a]Reagents and conditions: Case of **13j** a) 4-formylphenylboronic acid, $Pd(OAc)_2$, SPhos or $Pd(dppf)Cl_2$ CH_2Cl_2, K_3PO_4, 80°C, 79.5%,; b) 3,5-dichloroaniline, $NaBH(OAc)_3$, AcOH, CH_2Cl_2, r.t., 60.7%; c) 3-methyl-4-pyridinecarbonyl chloride hydrochloride, DIPEA, CH_2Cl_2, r.t. 28.3%.

S7. Supplementary Figure S4. Synthesis of *N*-substituted-phenyl-cycloalkyl-(3,5-dichlorophenyl)-3-methylisonicotinamide **20**.[a]

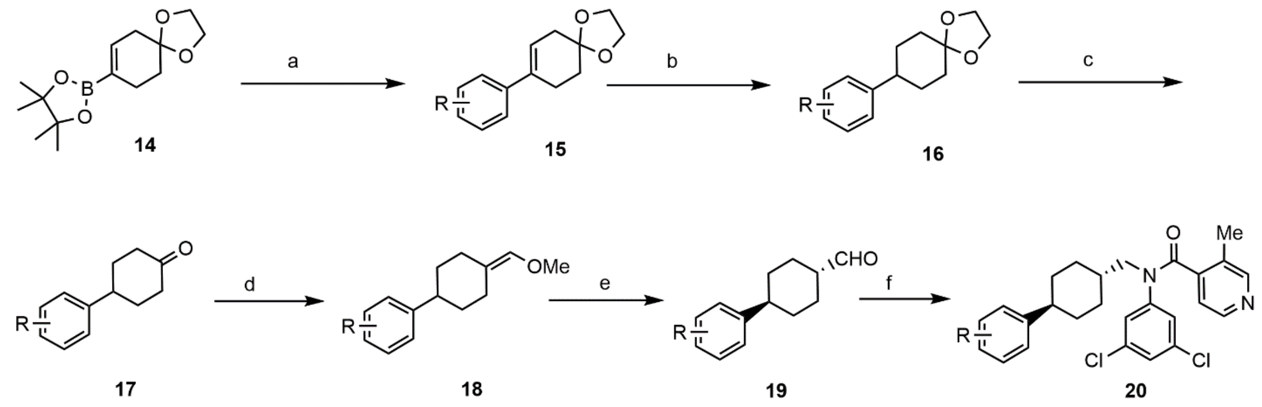

[a]Reagents and conditions: Case of **20d** a) substituted phenyl bromide, $Pd(OAc)_2$, SPhos or $Pd(dppf)Cl_2$ CH_2Cl_2, Na_2CO_3, 80°C, 56.2%; b) Pd-C/H_2, AcOEt, q. y; c) HCO_2H, H_2O, toluene, 120°C, 48.4%; d) NaHMDS, Ph_3PCH_2OMe THF, 64.7%; e) HCl/dioxane, 64.3%; f) 1) 3,5-dichloroaniline, $NaBH(OAc)_3$, AcOH, CH_2Cl_2, r.t., 80.0%; 2) 3-methyl-4-pyridinecarbonyl chloride hydrochloride, DIPEA, CH_2Cl_2, r.t., 15.3%.

S8. Supplementary Figure S5. Synthesis of *N*-substituted-aryl bicyclo[2,2,2]-octan-1-yl-methyl-(3,5-dichlorophenyl)-3-methylisonicotinamide **20** (route 1). [a]

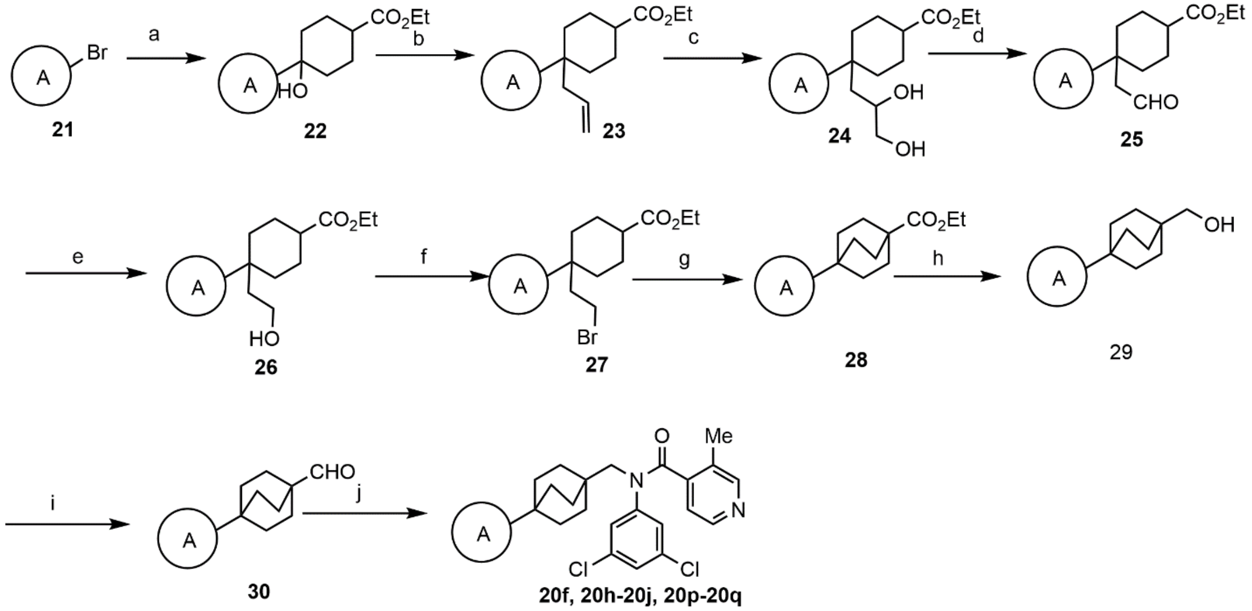

[a]Reagents and conditions. Case of **20p** a) ethyl 4-oxo-cyclohexyl carboxylate, *n*-BuLi, THF, -78°C, q.y; b) allyl trimethylsilane, BF_3 Et_2O, CH_2Cl_2, -78°C, 65.7%; c) OsO_4, *N*-methyl morphorine *N*-oxide, CH_3CN-H_2O, 0°C, q.y; d) $NaIO_4$, THF-H_2O, r.t. 70%; e) $NaBH_4$, THF, r.t. q.y; f) CBr_4, PPh_3, CH_2Cl_2, r.t.79.3%; g) LDA, HMPA, THF, -78°C, 56.5%; h) 1) 10% NaOH, MeOH, THF, 98.6%; 2) *i*-BuCOCl, Et_3N, THF; 3) $NaBH_4$, THF, H_2O, q.y.; i) SO_3-pyridine, DMSO, Et_3N, CH_2Cl_2, q.y.; j) 1) 3,5-dichloroaniline, $NaBH(OAc)_3$, AcOH, CH_2Cl_2, r.t. 23.7%; 2) 3-methyl-4-pyridinecarbonyl chloride hydrochloride, DIPEA, CH_2Cl_2, r.t. 85.9%

S9. Supplementary Figure S6. Synthesis of *N*-4-substituted-aryl bicyclo[2,2,2]-octan-1-yl-methyl- (3,5-dichlorophenyl)-3-methylisonicotinamide **20** (route 2). [a]

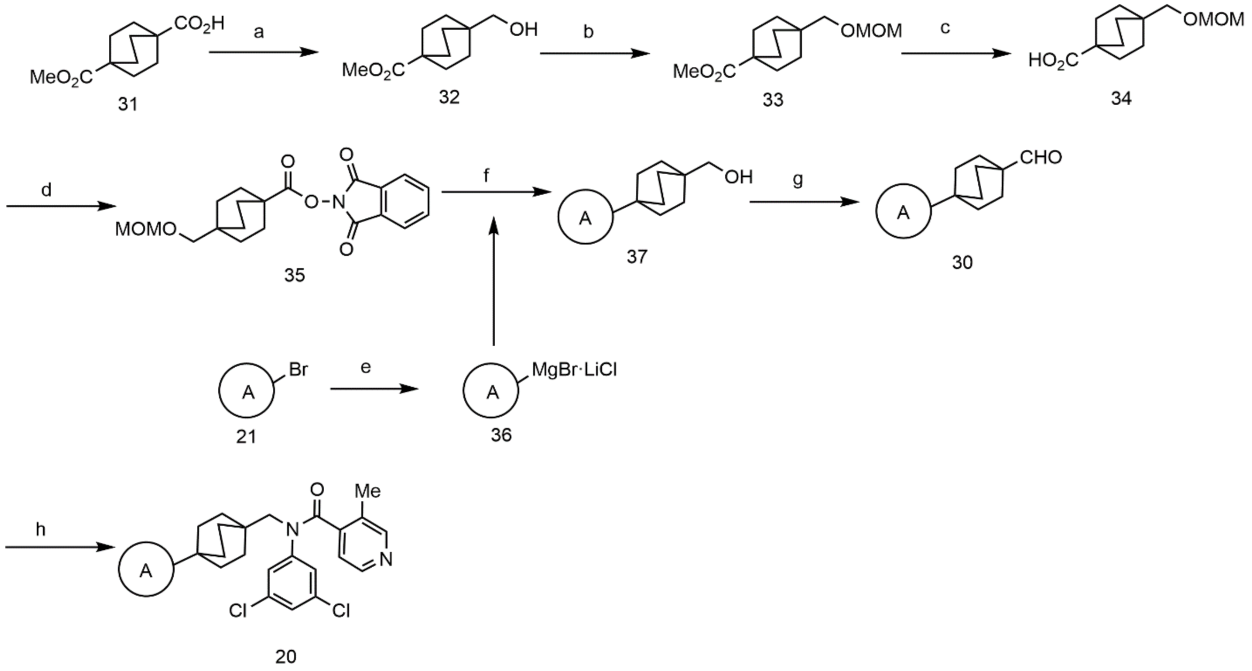

[a]Reagents and conditions: Case of **20r** a) MeSMe−BH_3 complex, CH_2Cl_2, 0°C, 88%; b) MOMCl, DIPEA, THF, r.t. 99%; c) 10% NaOH, MeOH, THF, r.t. q. y.; d) *N*-hydroxyphthalimide, DMAP, *N,N'*-disopropylcarbodiimide, CH_2Cl_2, r.t. 83%, ; e) LiCl, Mg, THF, cat-DIBAL; f) $Fe(acac)_3$, 1,3-dimethyl-3,4,5,6-tetrahydro-2(1*H*)-pyrimidinone, then 2N HCl-dioxane, r.t. 3steps 32%; g) SO_3-pyridine, DMSO, Et_3N, CH_2Cl_2, 76%; h) 1) 3,5-dichloroaniline, $NaBH(OAc)_3$, AcOH, CH_2Cl_2, r.t.; 2) 3-methyl-4-pyridinecarbonyl chloride hydrochloride, DIPEA, CH_2Cl_2, r.t., 95.0%

S10. Preparation of **13j**

The synthesis of typical compound **13j** was shown as below. Other compounds were synthesized as same procedure.

4'-{[(3,5-Dichlorophenyl)amino]methyl}-*N*,*N*-dimethyl-[1,1'-biphenyl]-4-amine (**12j**)

A mixture of 70 mg (0.3107 mmol) of 4'-(dimethylamino)-[1,1'-biphenyl]-4-carbaldehyde **11**[6], 355 mg (0.3418 mmol) of 3, 5-dichloroaniline, 200 mg (0.9321 mmol) of sodium triacetoxyborohydride, 56 mg (0.9321mmol) of acetic acid and 5ml of dichloromethane was stirred at room temperature for 18 hours. The solvent was evaporated. The residue was dissolved in water, and the aqueous layer was neutralized (pH=7) with 10% sodium hydroxide solution, and it was extracted with ethyl acetate (AcOEt). The organic layer was washed with brine, dried over sodium sulfate and concentrated under a vacuum to afford a crude product that was purified by silica gel column chromatography to afford 70 mg of the title compound **12j** as a colorless solid. Yield; 60.7 %.

^{1}H-NMR ($CDCl_3$) δ: 2.99 (6H, s), 4.17 (1H, br), 4.29 (2H, s), 6.51 (2H, s), 6.68 (1H, s), 6.80 (2H, d, J=1.5 Hz), 7.34 (2H, d, J=1.5 Hz), 7.49 (2H, d, J=1.5 Hz), 7.54 (2H, d, J=1.5 Hz).

***N*-(3,5-Dichlorophenyl)-*N*-{[4'-(dimethylamino)-[1,1'-biphenyl]-4-yl]methyl}-3-methylisonicotinamide** (**13j**)

A mixture of 70 mg (0.1885 mmol) of 4'-{[(3,5-dichlorophenyl)amino]methyl}-*N*,*N*-dimethyl-[1,1'-biphenyl]-4-amine, 100 mg (0.5655 mmol) of 3-methyl-4-pyridinecarbonyl chloride hydrochloride, 0.24 g (1.885 mmol) of *N*,*N*-diisopropylethylamine and 5 ml of dichloromethane was stirred at room temperature for 18 hours. The reaction mixture was quenched with water and extracted with dichloromethane. The organic layer was washed with brine, dried over sodium sulfate, filtered and evaporated under a vacuum to afford a crude product that was purified by silica gel column chromatography to afford 50 mg of compound **13j** as a pale-yellow solid. Yield; 28.3 %. ^{1}H-NMR ($CDCl_3$) δ :2.34 (3H, s), 2.99 (6H, s), 5.08 (2H, s), 6.79 (3H, brd, J=1.5 Hz), 6.93 (2H, br), 7.13 (1H, br), 7.30 (3H, br), 7.45-7.58 (4H, m), 8.31 (1H, br), 8.40 (1H, br)., ^{13}C-NMR (DMSO-d_6) δ:, 15.9, 40.0, 51.1, 112.6, 121.2, 125.6, 126.9, 127.0, 127.2, 127.3, 128,7, 133.9, 134.0, 139.5, 143.0, 143.1, 146.8, 149.9, 151.2, 167.5., m/z: 490.20 $(M+H)^+$, purity; > 99.9% (2.73 min), Method A, HRMS (ESI) calculated: 490.14474 $(M+H)^+$, found 490.14465.
Compounds **13e-g**, **13k-l**, **13p-r**, **13v-ah** were synthesized according to the same procedure as shown in the synthesis of compound **13j**.

S11. NMR and LCMS data of **13e-g**, **13k-l**, **13p-r**, **13v-ah**.

Supplementary Table S2. NMR and LCMS data of **13e-g**, **13k-l**, **13p-r**, **13v-ah**.

Ex.	Structure	NMR data	Mass m/z:$(M+H)^+$
**13e**	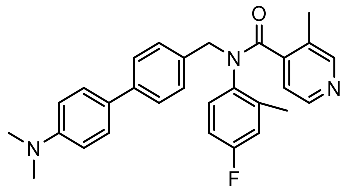	^{1}H-NMR ($CDCl_3$) δ: 2.15 (3H, s), 2.35 (3H, s), 3.00 (6H, s), 4.40 (1H, d, J=13.6 Hz), 5.55 (1H, d, J=13.6 Hz), 6.54-6.67 (2H, m), 6.73-6.81 (2H, m), 6.80 (2H, d, J=9.2 Hz), 7.27 (2H, d, J=9.2 Hz), 7.49 (2H, d, J=8.8 Hz), 7.51 (2H, d, J=8.8 Hz), 8.16 (1H, d, J=4.4 Hz), 8.33 (1H, s).	454.19<br>95.7%<br>(2.18 min)<br>Method A
**13f**	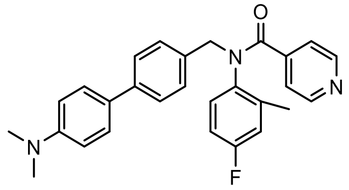	^{1}H-NMR ($CDCl_3$) δ: 1.94 (3H, s), 2.99 (6H, s), 4.63 (1H, d, J=13.6 Hz), 5.33 (1H, d, J=13.6 Hz), 6.69-6.79 (2H, m), 6.80 (2H, d, J=8.8 Hz), 6.84 (1H, dd, J=8.4, 5.6 Hz), 7.12 (2H, d, J=6.0 Hz), 7.26 (2H, d, J=8.4 Hz), 7.48 (2H, d, J=8.4 Hz), 7.50 (2H, d, J=8.8 Hz), 8.44 (2H, d, J=6.0 Hz).	440.26<br>99.1%<br>(2.33 min)<br>Method A
**13g**	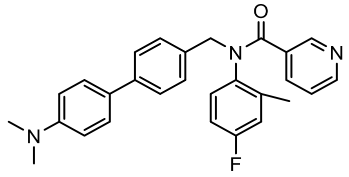	^{1}H-NMR ($CDCl_3$) δ: 1.92 (3H, s), 2.99 (6H, s), 4.68 (1H, d, J=13.6 Hz), 5.31 (1H, d, J=13.6 Hz), 6.69-6.78 (2H, m), 6.79 (2H, d, J=9.2 Hz), 6.89 (1H, dd, J=9.2, 5.2 Hz), 7.10 (1H, dd, J=8.0, 4.8 Hz), 7.27 (2H, d, J=9.2 Hz), 7.48 (2H, d, J=8.0 Hz), 7.49 (2H, d, J=8.0 Hz), 7.62 (1H, dt, J=8.0, 1.6 Hz), 8.45 (1H, dd, J=4.8, 1.6 Hz), 8.50 (1H, d, J=1.6 Hz).	440.26<br>95.3%<br>(2.41 min)<br>Method A
**13k**	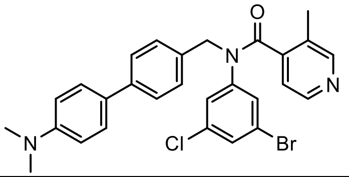	^{1}H-NMR ($CDCl_3$) δ: 2.34 (3H, s), 3.00 (6H, s), 5.08 (2H, brs), 6.80 (3H, d, J=8.5 Hz), 6.89-7.00 (2H, br), 7.25-7.35 (3H, m), 7.52 (4H , t, J=8.5 Hz), 8.20-8.35 (1H, brd), 8.35-8.45 (1H, m).	536.02<br>95.1%<br>(3.22 min)<br>Method A
**13l**	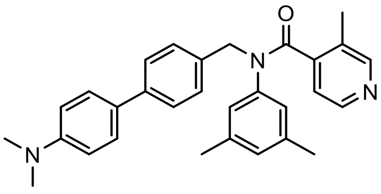	^{1}H-NMR ($CDCl_3$) δ:2.10 (6H, s), 2.33 (3H, s), 3.00 (6H, s), 5.08 (2H, s), 6.48 (2H, s), 6.72 (1H, s), 6.80 (2H, d, J=8.5 Hz), 6.92 (1H, d, J=5.5 Hz), 7.32 (2H, d, J=8.5 Hz), 7.51 (4H, d, J=8.5 Hz), 8.21 (1H, d, J=5.5 Hz), 8.31 (1H, s).	450.27<br>>99.9%<br>(2.19 min)<br>Method A
**13p**	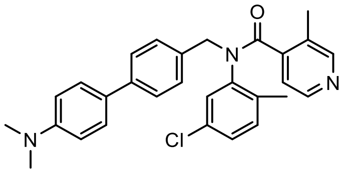	^{1}H-NMR ($CDCl_3$) δ: 2.04 (3H, s), 2.37 (3H, s), 3.00 (6H, s), 4.61 (1H, d, J=14.0 Hz), 5.38 (1H, d, J=14.0 Hz), 6.75 (1H, d, J=2.0 Hz), 6.80 (1H, d, J=4.8 Hz), 6.80 (2H, d, J=9.2 Hz), 6.99 (1H, d, J=8.4 Hz), 7.06 (1H, dd, J=8.4, 2.0 Hz), 7.28 (2H, d, J=8.0 Hz), 7.50 (2H, d, J=8.0 Hz), 7.51 (2H, d, J=9.2 Hz), 8.17 (1H, d, J=4.8 Hz), 8.36 (1H, s).	470.23<br>95.3%<br>(2.47 min)<br>Method B

Supplementary Table S2. NMR and LCMS data of **13e-g**, **13k-l**, **13p-r**, **13v-ah**. (continue)

Ex.	Structure	NMR data	Mass m/z:(M+H)$^{+}$
**13q**	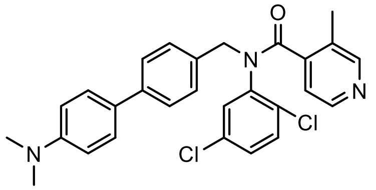	^{1}H-NMR ($CDCl_3$) δ: 2.39 (3H, s), 3.00 (6H, s), 4.28 (1H, d, J=14.0 Hz), 5.80 (1H, d, J=14.0 Hz), 6.63 (1H, d, J=2.4 Hz), 6.81 (2H, d, J=8.4 Hz), 7.00 (1H, d, J=4.8 Hz), 7.09 (1H, dd, J=8.4, 2.4 Hz), 7.27 (1H, d, J=8.4 Hz), 7.28 (2H, d, J=8.4 Hz), 7.51 (4H, d, J=8.4 Hz), 8.22 (1H, d, J=4.8 Hz), 8.38 (1H, s).	490.00<br>95.9%<br>(2.83 min)<br>Method B
**13r**	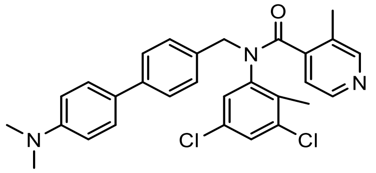	^{1}H-NMR ($CDCl_3$) δ: 2.11 (3H, s), 2.36 (3H, s), 3.00 (6H, s), 4.50 (1H, d, J=14.0 Hz), 5.45 (1H, d, J=13.6 Hz), 6.63 (1H, d, J=1.6 Hz), 6.76 (3H, m), 7.22 (1H, d, J=2.0 Hz), 7.24-7.29 (2H, m), 7.52 (4H, d, J=8.8 Hz), 8.22 (1H, d, J=4.8 Hz), 8.38 (1H, s).	504.31<br>95.7%<br>(2.01 min)<br>Method B
**13v**	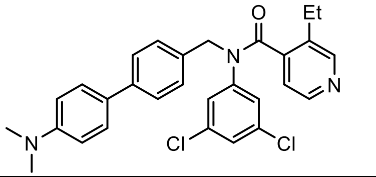	^{1}H-NMR ($CDCl_3$) δ: 1.31 (3H, d, J=7.6 Hz), 2.70 (2H, q, J=7.2 Hz), 3.00 (6H, s), 5.08 (2H, s), 6.73-6.84 (4H, m), 6.89 (1H, s), 7.12 (1H, s), 7.30 (2H, d, J=7.6 Hz), 7.52 (4H, t, J=7.2 Hz) 8.29 (1H,s), 8.46 (1H, s).	504.31<br>96.1%<br>(2.01 min)<br>Method B
**13w**	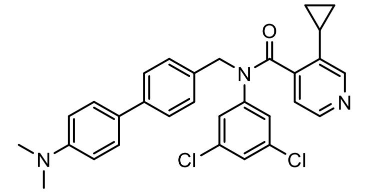	^{1}H-NMR ($CDCl_3$) δ: 0.83 (2H, s), 1.10 (2H, d, J=7.6 Hz), 1.92-2.02 (1H, m), 3.00 (6H, s), 5.10 (2H, s), 6.81 (2H, d, J=9.2 Hz), 6.82 (2H, s), 6.92 (1H, d, J=4.4 Hz), 7.11 (1H, s), 7.31 (2H, d, J=7.6 Hz), 7.52 (4H, t, J=6.8 Hz) 8.03 (1H,s), 8.26 (1H, d, J=5.2 Hz).	516.39<br>95.3%<br>(2.02 min)<br>Method B
**13x**	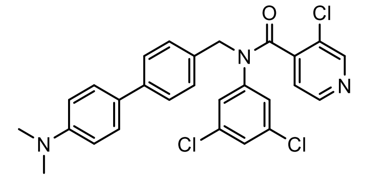	^{1}H-NMR ($CDCl_3$) δ: 3.00 (6H, s), 5.08 (2H, s), 6.80 (2H, d, J=8.5 Hz), 6.90 (2H, d, J=1.5 Hz), 7.09 (1H, d, J=4.5 Hz), 7.15 (1H, t, J=1.5 Hz), 7.31 (2H, d, J=8.5 Hz), 7.53 (4H, d, J=8.5 Hz), 8.39 (1 H, d, J=5.0 Hz), 8.50 (1H, s).	510.06<br>95.9%<br>(2.62 min)<br>Method B
**13y**	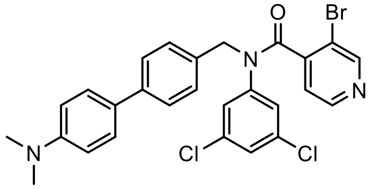	^{1}H-NMR ($CDCl_3$) δ: 2.99 (6H, s), 5.07 (2H, s), 6.80 (2H, d, J=8.0 Hz), 6.93 (2H, d, J=2.0 Hz), 7.05 (1H, d, J=5.2 Hz), 7.14 (1H, t, J=1.6 Hz), 7.31 (2H, d, J=8.0 Hz), 7.51 (4H, t, J=8.0 Hz) 8.40 (1H, d, J=4.8 Hz), 8.62 (1H, s).	556.23<br>95.8%<br>(2.26 min)<br>Method B
**13z**	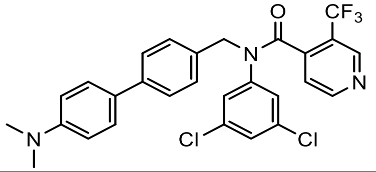	^{1}H-NMR ($CDCl_3$) δ: 2.99 (6H, s), 5.07 (2H, brs), 6.79 (2H, d, J=8.8 Hz), 6.81-6.86 (1H, br), 7.11 (2H, brd, J=4.4 Hz), 7.10-7.15 (1H, br), 7.24-7.30 (2H, br), 7.50 (4H, t, J=8.8 Hz), 8.61 (1H, d, J=4.4 Hz), 8.42 (1H, s).	544.05<br>>99.9%<br>(2.63 min)<br>Method B

Supplementary Table S2. NMR and LCMS data of **13e-g**, **13k-l**, **13p-r**, **13v-ah**. (continue)

Ex.	Structure	NMR data	Mass m/z:(M+H)+
**13aa**	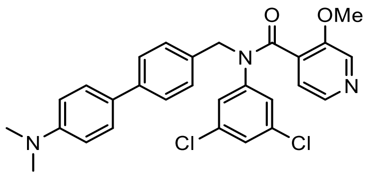	^{1}H-NMR ($CDCl_3$) δ: 2.99 (6H, s), 3.84 (3H, s), 5.04 (2H, brs), 6.79 (2H, d, J=8.8 Hz), 6.81-6.96 (2H, br), 7.11 (2H, brd, J=3.2 Hz), 7.21-7.37 (2H, br), 7.49 (2H, d, J=7.6 Hz), 7.51 (2H, d, J=7.6 Hz), 8.10-8.26 (2H, m).	506.05<br>96.4%<br>(2.07 min)<br>Method B
**13ab**	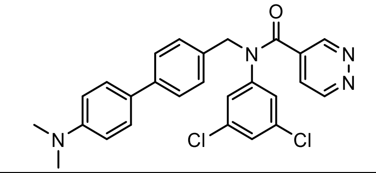	^{1}H-NMR ($CDCl_3$) δ: 3.00 (6H, s), 5.08 (2H, s), 6.80 (2H, d, J=8.8 Hz), 6.87 (2H, brs), 7.23-7.25 (1H, m), 7.27-7.29 (1H, m), 7.41 (1H, dd, J=4.8, 2.0 Hz), 7.48-7.54 (5H, m), 9.05 (1H, s), 9.19 (1H, dd, J=4.8, 0.8 Hz)	477.10<br>>99.9%<br>(2.37 min)<br>Method B
**13ac**	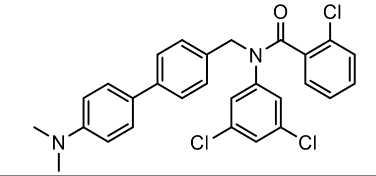	^{1}H-NMR ($CDCl_3$) δ: 3.00 (6H, s), 5.10 (2H, s), 6.80 (2H, d, J=8.5 Hz), 6.91 (2H, s), 7.07 (1H, s), 7.14-7.20 (3H, m), 7.25-7.30 (1H, m), 7.32-7.38 (2H, m), 7.52 (4H, t, J=8.5 Hz).	509.00<br>>99.9%<br>(4.19 min)<br>Method A
**13ad**	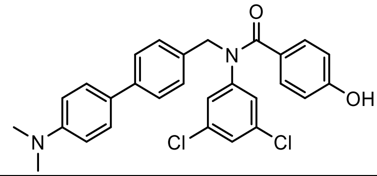	^{1}H-NMR ($CDCl_3$) δ: 2.99 (6H, s), 5.05 (1H, s), 5.09 (2H, s), 6.67-6.70 (2H, m), 6.79 (2H, d, J=8.8 Hz), 6.87 (2H, d, J=2.0 Hz), 7.12 (1H, t, J=1.6 Hz), 7.29-7.31 (4H, m), 7.48-7.52 (4H, m)	491.02<br>>99.9%<br>(2.63 min)<br>Method B
**13ae**	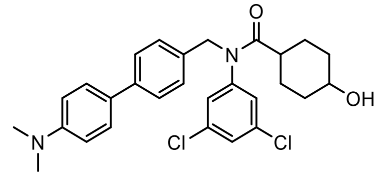	^{1}H-NMR ($CDCl_3$) δ: 1.00-1.10 (2H, m), 1.33-1.36 (1H, m), 1.65-1.78 (3H, m), 1.95-2.15 (3H, m), 3.00 (6H, s), 3.55-3.67 (1H, m), 4.83 (2H, s), 6.80 (2H, d, J=8.5 Hz), 6.92 (2H, s), 7.17 (2H, d, J=7.5 Hz), 7.35-7.38 (1H, m), 7.54 (4H, t, J=8.5 Hz).	497.06<br>>99.9%<br>(2.63 min)<br>Method B
**13af**	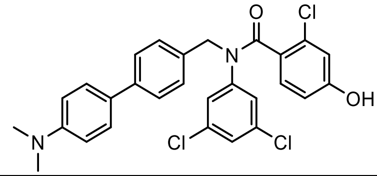	^{1}H-NMR (DMSO-d_6) δ: 2.93 (6H, s), 5.09 (2H, brs), 6.55-6.73 (2H, m), 6.77 (2H, d, J=9.0 Hz), 7.19-7.28 (3H, m), 7.29 (2H, d, J=8.5 Hz), 7. 38 (1H, s), 7.50 (2H, d, J=9.0 Hz), 7.55 (2H, d, J=8.5 Hz).	525.05<br>97.0%<br>(2.84 min)<br>Method B
**13ag**	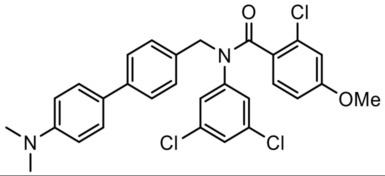	^{1}H-NMR (DMSO-d_6) δ: 2.93 (6H, s), 3.74 (3H, s), 5.13 (2H, brs), 6.78 (2H, d, J=8.4 Hz), 6.86-6.88 (1H, m), 6.99 (1H, br s), 7.24-7.33 (4H, m), 7.40-7.45 (2H, m), 7.51 (2H, d, J=8.8 Hz), 7.55 (2H, d, J=8.4 Hz).	541.00<br>97.0%<br>(3.75 min)<br>Method B
**13ah**	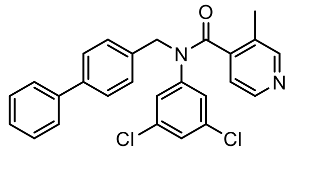	^{1}H-NMR ($CDCl_3$) δ: 2.35 (3H, s), 5.11 (2H, s), 6.70-6.95 (3H, br), 7.15 (1H, br), 7.36 (3H, br), 7.45 (2H, t, J=7.2 Hz), 7.56 (4H, t, J=7.2 Hz), 8.30-8.45 (2H, br).	446.98<br>95.9%<br>(2.58 min)<br>Method B

S12. Preparation of **13m** (Heck reaction)

The synthesis procedure of typical compound **13m** showed below. Other compounds were synthesized using same procedure.

A mixture of 20 mg (0.03739 mmol) of **13k**, 54 mg (0.7478 mmol) of methyl acrylate, 9 mg (0.01121 mmol) of palladium (II) acetate, 21 mg (0.02242 mmol) of tri(*o*-tolyl) phosphine, 72 mg (0.2356 mmol) of triethylamine and 5 ml of acetonitrile was stirred at 80°C for 18 hours. The reaction mixture was quenched with water and extracted with AcOEt. The organic layer was washed with brine, dried over sodium sulfate, filtered and evaporated under a vacuum to afford a crude product that was purified by silica gel column chromatography to yield 5 mg of the title compound **13m** as a pale brown amorphous. Yield; 25 %.

^{1}H-NMR ($CDCl_3$) δ: 2.38 (3H, s), 3.00 (6H, s), 3.78 (3H, s), 5.11 (2H, s), 6.18 (1H, d, J=16.0 Hz), 6.78-6.84 (3H, m), 6.92 (2H, br), 7.20-7.74 (4H, m), 7.45-7.56 (4H, m), 8.21 (1H, brd, J=4.0 Hz), 8.37 (1H, s)., m/z: 540.08 $(M+H)^+$, purity; >99.9% (2.40 min), Method B.

Compounds **13a-d**, **13h-i**, **13u** were synthesized according to the same procedure as shown in the synthesis of compound **13m**.

S13. NMR and LCMS data of **13a-d**, **13h-i**, **13u**

Supplementary Table S3. NMR and LCMS data of **13a-d**, **13h-i**, **13u**.

Ex.	Structure	NMR data	Mass m/z:$(M+H)^+$
**13a**	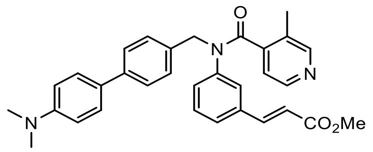	^{1}H-NMR ($CDCl_3$) δ: 2.34 (3H, s), 2.99 (6H, s), 3.78 (3H, s), 5.12 (2H, s), 6.20 (1H, d, J=16.0 Hz), 6.80 (2H, d, J=8.4 Hz), 6.86 (1H, brd, J=7.6 Hz), 6.92 (1H, brd, J=4.8 Hz), 7.01 (1H, s), 7.12 (1H, t, J=7.6 Hz), 7.26 (1H, brd, J=7.6 Hz), 7.31 (2H, d, J=7.6 Hz), 7.45 (1H, d, J=16.0 Hz), 7.50 (2H, d, J=8.4 Hz), 7.51 (2H, d, J=7.6 Hz), 8.21 (1H, brd, J=4.8 Hz), 8.32 (1H, s).	506.34<br>95.3%<br>(2.45 min)<br>Method A
**13b**	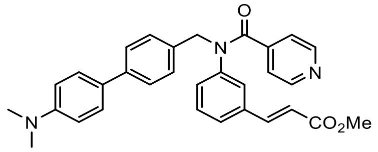	^{1}H-NMR ($CDCl_3$) δ: 2.99 (6H, s), 3.78 (3H, s), 5.12 (2H, s), 6.24 (1H, d, J=16.0 Hz), 6.79 (2H, d, J=8.8 Hz), 6.92 (1H, brd, J=8.0 Hz), 7.07 (1H, brs), 7.18 (2H, d, J=6.0 Hz), 7.19 (1H, t, J=8.0 Hz), 7.29 (2H, d, J=8.0 Hz), 7.32 (1H, d, J=8.0 Hz), 7.48 (1H, d, J=16.0 Hz), 7.49 (2H, d, J=8.8 Hz), 7.50 (2H, d, J=8.0 Hz), 8.47 (2H, d, J=6.0 Hz).	492.28<br>97.1%<br>(2.21 min)<br>Method A
**13c**	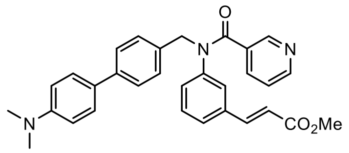	^{1}H-NMR ($CDCl_3$) δ: 2.99 (6H, s), 3.77 (3H, s), 5.15 (2H, s), 6.20 (1H, d, J=16.0 Hz), 6.79 (2H, d, J=9.2 Hz), 6.95 (1H, brd, J=7.6 Hz), 7.08 (1H, s), 7.14 (1H, dd, J=8.0, 4.8 Hz), 7.20 (1H, t, J=7.6 Hz), 7.30 (2H, d, J=8.4 Hz), 7.31 (1H, d, J=7.6 Hz), 7.48 (1H, d, J=16.0 Hz), 7.49 (2H, d, J=8.4 Hz), 7.49 (2H, d, J=9.2 Hz), 7.68 (1H, dt, J=8.0, 1.6 Hz), 8.46 (1H, dd, J=4.8, 1.6 Hz), 8.53 (1H, d, J=1.6 Hz).	492.32<br>95.2%<br>(2.30 min)<br>Method A
**13d**	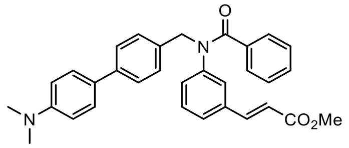	^{1}H-NMR ($CDCl_3$) δ: 2.99 (6H, s), 3.77 (3H, s), 5.14 (2H, s), 6.23 (1H, d, J=16.0 Hz), 6.79 (2H, d, J=9.2 Hz), 6.95 (1H, brd, J=7.6 Hz), 7.08 (1H, s), 7.13-7.20 (3H, m), 7.21-7.26 (2H, m), 7.30-7.36 (4H, m), 7.45-7.51 (5H, m)	491.31<br>95.0%<br>(3.06 min)<br>Method A
**13h**	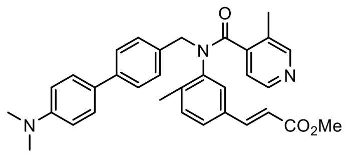	^{1}H-NMR($CDCl_3$) δ: 2.15 (3H, s), 2.38 (3H, s), 3.00 (6H, s), 3.76 (3H, s), 4.50 (1H, d, J=13.6 Hz), 5.51 (1H, d, J=14.0 Hz), 6.12 (1H, d, J=16.0 Hz), 6.76-6.85 (4H, m), 7.10 (1H, d, J=8.0 Hz), 7.21-7.31 (2H, m), 7.38 (1H, d, J=15.6 Hz), 7.47-7.53 (4H, d, J=8.8 Hz), 8.05 (1H, s), 8.15 (1H, d, J=5.2 Hz), 8.33 (1H, s).	520.29<br>97.5%<br>(1.50 min)<br>Method B

Supplementary Table S3. NMR and LCMS data of **13a-d**, **13h-i**, **13u**. (continue)

Ex.	Structure	NMR data	Mass m/z:$(M+H)^+$
**13i**	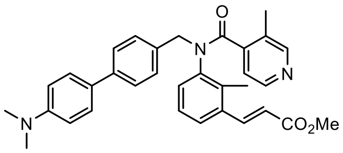	^{1}H-NMR ($CDCl_3$) δ: 2.26 (3H, s), 2.36 (3H, s), 3.00 (6H, s), 3.80 (3H, s), 4.38 (1H, d, J=13.6 Hz), 5.61 (1H, d, J=13.6 Hz), 6.27 (1H, d, J=16.0 Hz), 6.67 (1H, d, J=7.6 Hz), 6.76 (1H, d, J=5.2 Hz), 6.80 (2H, d, J=8.8 Hz), 6.92 (1H, t, J=7.6 Hz), 7.28 (2H, d, J=8.0 Hz), 7.35 (1H, d, J=7.6 Hz), 7.49 (2H, d, J=8.0 Hz), 7.51 (2H, d, J=8.8 Hz), 7.81 (1H, d, J=16.0 Hz), 8.13 (1H, d, J=5.2 Hz), 8.32 (1H, s).	520.21<br>95.5%<br>(2.80 min)<br>Method A
**13u**	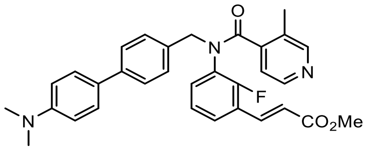	^{1}H-NMR ($CDCl_3$) δ: 2.37 (3H, s), 3.00 (6H, s), 3.81 (3H, s), 4.55 (1H, d, J=14.4 Hz), 5.61 (1H, d, J=14.4 Hz), 6.41 (1H, d, J=16.0 Hz), 6.78 (1H, t, J=6.8 Hz), 6.79 (2H, d, J=8.8 Hz), 6.87 (1H, t, J=8.0 Hz), 6.95 (1H, d, J=5.2 Hz), 7.23-7.37 (3H, m), 7.48 (2H, s), 7.51 (2H, d, J=1.6 Hz), 7.65 (1H, d, J=16.4 Hz), 8.21 (1H, d, J=4.8 Hz), 8.34 (1H, s).	524.16<br>95.9%<br>(1.48 min)<br>Method B

S14. Preparation of **13n** (Buchwald reaction)

A mixture of 46 mg (0.08226 mmol) of **13k**, 10 mg (0.09048 mmol) of azetidine, 84 mg (0.2468 mmol) of cesium carbonate, 6 mg (0.01176 mmol) of 4,5-bis(diphenylphosphino)-9,9-dimethylxathene, 6 mg (0.005758 mmol) of tris(dibenzylideneacetone)dipalladium (0) and 5 ml of dioxane was stirred at 100°C for 18 hours under an argon atmosphere. The reaction mixture was quenched with water and extracted with AcOEt. The organic layer was washed with brine, dried over sodium sulfate, filtered and evaporated under a vacuum to afford a crude product that was purified by silica gel column chromatography to provide 30 mg of the title compound **13n** as a pale brown amorphous. Yield; 59 %. ^{1}H-NMR ($CDCl_3$) δ: 2.27 (2H, t, J=7.5 Hz), 2.34 (3H, s), 3.00 (6H, s), 3.61 (4H, t, J=7.5 Hz), 5. 06 (2H, s), 5.60 (1H, s), 6.08 (1H, s), 6.30 (1H, s), 6.80 (2H, d, J=9.0 Hz), 6.95 (1H, d, J=4.5 Hz), 7.33 (2H, d, J=7.5 Hz), 7.40-7.44 (1H, m), 7.49-7.54 (3H, m), 8.27 (1H, d, J=4.0 Hz), 8.35 (1H, s)., m/z: 511.18 $(M+H)^+$, purity; 95.6% (2.65 min), Method B.

S15. Preparation of **13o** (Suzuki reaction)

The synthesis procedure of typical compound **13o** was shown below. Other compounds were synthesized by same procedure of compound **13o**.

A mixture of 66 mg of **13k**, 44 mg of 1-(propan-2-yl)-4-(4,4,5,5-tetramethy-1,3,2-dioxoboran-2-yl)-1*H*-pyrazole, 55 mg of sodium carbonate, 10 mg of [1,1'-bis(diphenylphosphino)ferrocene]palladium (II) dichloride dichloromethane adduct, 4 ml of dioxane and 1 ml of water was stirred at 80°C for 18 hours under an argon atmosphere. The reaction mixture was quenched with water and extracted with AcOEt through a Celite filter. The organic layer was washed with brine, dried over sodium sulfate, filtered and evaporated under a vacuum to afford a crude product that was purified by silica gel column chromatography to yield the title compound **13o** as a pale brown amorphous. Yield; 56 %. ^{1}H-NMR ($CDCl_3$) δ: 1.48 (6H, d, J=6.8 Hz), 2.36 (3H, s), 3.00 (6H, s), 4.37-4.50 (1H, m), 5.12 (2H, s), 6.74 (2H, s), 6.80 (2H, d, J=8.8 Hz), 6.97 (1H, d, J=5.2 Hz), 7.18 (1H, s), 7.34 (1H, s) 7.36 (2H, d, J=5.2 Hz), 7.43-7.58 (5H, m), 8.26 (1H, d, J=4.8 Hz), 8.36 (1H, s). MASS m/z: 564.23, purity; 96.5% (1.73 min) Method B.

Compounds **13s-t** were synthesized according to the same procedure as shown in the synthesis of compound **13o**.

S16. NMR and LCMS data of **13s-t**

Supplementary Table S4. NMR and LCMS data of **13s-t**.

Ex.	Structure	NMR data	Mass m/z:(M+H)+
**13s**	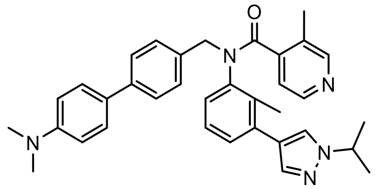	^{1}H-NMR ($CDCl_3$) δ: 1.55 (6H, d, J=6.8 Hz), 2.25 (3H, s), 2.37 (3H, s), 3.00 (6H, s), 4.38 (1H, d, J=13.6 Hz), 4.52 (1H, sept, J=6.8 Hz), 5.65 (1H, d, J=13.6 Hz), 6.56 (1H, dd, J=8.0, 1.2 Hz), 6.80 (2H, d, J=8.8 Hz), 6.80 (1H, d, J=5.2 Hz), 6.87 (1H, t, J=8.0 Hz), 7.13 (1H, dd, J=8.0, 1.2 Hz), 7.32 (2H, d, J=8.4 Hz), 7.42 (1H, s), 7.50 (2H, d, J=8.4 Hz), 7.51 (2H, d, J=8.8 Hz), 7.53 (1H, s), 8.14 (1H, d, J=5.2 Hz), 8.33 (1H, s).	544.38<br>97.2%<br>(2.77 min)<br>Method B
**13t**	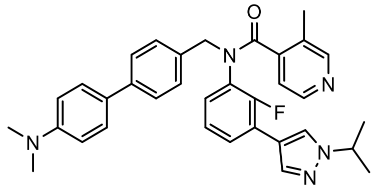	^{1}H-NMR ($CDCl_3$) δ: 1.55 (6H, d, J=6.4 Hz), 2.38 (3H, s), 2.99 (6H, s), 4.45-4.60 (1H, m), 4.56 (1H, d, J=14.4 Hz) 5.63 (1H, d, J=14.4 Hz), 6.56 (1H, td, J=1.2, 8.0 Hz), 6.75-6.85 (3H, m), 7.01 (1H, d, J=5.2 Hz), 7.25-7.36 (1H, m), 7.31 (2H, d, J=8.0 Hz), 7.49 (4H, d, J=8.8 Hz), 7.69 (1H, d, J=1.6 Hz), 7.76 (1H, d), 8.20 (1H, d, J=4.8 Hz), 8.34 (1H, s).	548.33<br>95.8%<br>(1.50 min)<br>Method B

S17. Preparation of **20d**.

3,5-Dichloro-*N*-({[(1,4-*trans*)-4-[4-methoxy-3-methylphenyl]cyclohexyl}methyl)aniline

A mixture of 64 mg of 1,4-*trans*-4-(4-methoxy-3-methylphenyl)cyclohexanecarbaldehyde **19d** [7], 145 mg of 3,5-dichloroaniline, 50 mg of sodium triacetoxyborohydride, 50 mg of acetic acid and 3 ml of dichloromethane was stirred at room temperature for 18 hours, and the solvent was evaporated. The residue was dissolved in water. The aqueous layer was neutralized (pH=7) with 10% sodium hydroxide solution, and it was extracted with AcOEt. The organic layer was washed with brine, dried over sodium sulfate, and concentrated under a vacuum to afford a crude product that was purified by silica gel column chromatography to yield 74 mg of the title compound as a colorless solid. Yield; 80.0 %.

***N*-3,5-Dichlorophenyl-*N*-({[(1,4-*trans*)-4-[4-methoxy-3-methylphenyl]cyclohexyl}methyl)-3-methylisonicotinamide (20d)**

A mixture of 74 mg of 3,5-dichloro-*N*-({[(1,4-*trans*)-4-[4-methoxy-3-methylphenyl]cyclohexyl}methyl)aniline, 113 mg of 3-methyl-4-pyridinecarbonyl chloride hydrochloride, 0.40 g of *N*,*N*-diisopropylethylamine, 24 mg of 4-dimethylaminopyridine and 4 ml of dichloromethane was stirred at room temperature for 18 hours. The reaction mixture was quenched with water and extracted with dichloromethane. The organic layer was washed with brine, dried over sodium sulfate, filtered and evaporated under a vacuum to afford a crude product that was purified by silica gel column chromatography to afford 19 mg of the title compound as a pale brown amorphous. Yield; 15.3 %. ^{1}H-NMR ($CDCl_3$) δ: 1.09-1.31 (2H, m), 1.32-1.48 (2H, m), 1.54-1.75 (1H, m), 1.78-2.01 (4H, m), 2.19 (3H, s), 2.27-2.46 (1H, m), 2.33 (3H, s), 3.63-3.97 (2H, m), 3.79 (3H, s), 6.73 (1H, d, J=8.8 Hz), 6.81-7.08 (5H, m), 7.19 (1H, brs), 8.32 (1H, brs), 8.42 (1H, brs)., ^{13}C-NMR (DMSO-d_6) δ: 15.9, 16.1 ,30.6, 33.6, 35.4, 42.7, 54.0, 55.1, 110.0, 121.1, 124.8, 125.1, 126.8, 127.3, 128.7, 128.8, 134.1, 138.7, 143.4, 143.9, 146.7, 151.1, 155.5, 167.5., m/z: 497.11$(M+H)^+$, purity ; 96.9%, (3.15 min) Method A, HRMS (ESI) calculated: 497.17571 $(M+H)^+$, found 497.17566.

Compounds **20a-c**, **20e** were synthesized according to the same procedure as shown in the synthesis compound **20d**.

S18. NMR and LCMS data of **20a-c**, **20e**.

Supplementary Table S5. NMR and LCMS data of **20a-c, 20e**.

Ex.	Structure	NMR data	Mass m/z:(M+H)$^+$
**20a**	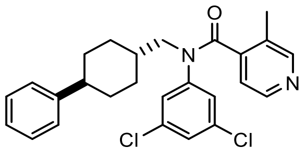	^{1}H-NMR ($CDCl_3$) δ: 1.20-1.36 (2H, m), 1.36-1.54 (2H, m), 1.68 (1H, brs), 1.84-2.03 (4H, m), 2.36 (3H, s), 2.51 (1H, s), 3.88 (2H, d, J=5.2 Hz), 6.89 (1H, s), 6.94 (2H, s), 7.11-7.23 (4H, m), 7.29 (2H, t, J=8.0 Hz), 8.31 (1H, brs), 8.41 (1H, brs).	453.36<br>95.1%<br>(2.36 min)<br>Method B
**20b**	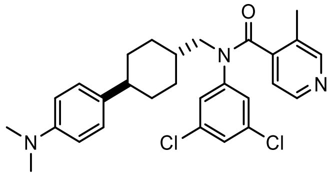	^{1}H-NMR ($CDCl_3$) δ: 1.09-1.29 (2H, m), 1.31-1.45 (2H, m), 1.50-1.74 (1H, m), 1.80-1.99 (4H, m), 2.28-2.46 (1H, m), 2.33 (3H, s), 2.90 (6H, s), 3.59-4.08 (2H, m), 6.68 (2H, d, J=8.8 Hz), 6.82-6.93 (1H, br), 6.93-7.02 (2H, br), 7.05 (2H, d, J=8.8 Hz), 7.18 (1H, brs), 8.32 (1H, brs), 8.41 (1H, brs)., 15.9, 30.6, 33.6, 35.5, 40.5, 42.6, 54.1, 112.7, 121.2, 126.8, 127.0, 127.3, 128.7, 134.1, 135.1, 143.5, 144.0, 146.7, 148.9, 151.1, 167.4.	496.09<br>95.4%<br>(2.07 min)<br>Method B
**20c**	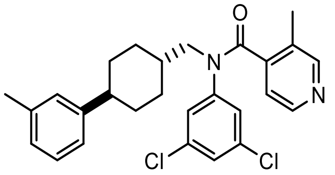	^{1}H-NMR ($CDCl_3$) δ: 1.18-1.34 (2H, m), 1.35-1.52 (2H, m), 1.56-1.74 (1H, m), 1.78-2.01 (4H, m), 2.33 (3H, s), 2.35 (3H, s), 2.41-2.54 (1H, m), 3.72-4.01 (2H, m), 6.80-7.06 (6H, m), 7.12-7.23 (2H, m), 8.30 (1H, brs), 8.40 (1H, brs).	467.09<br>95.5%<br>(2.62 min)<br>Method B
**20e**	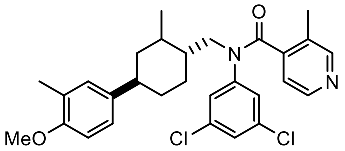	^{1}H-NMR ($CDCl_3$) δ: 0.94 (3H, br), 1.05-1.40 (5H, m), 1.75-2.00 (3H, m), 2.21 (3H, s), 2.33 (3H, s), 2.40-2.53 (1H, m), 3.79 (3H, s), 3.90-3.98 (2H, m), 6.70-6.75 (1H, m), 6.80-7.00 (5H, m), 7.18 (1H, br), 8.31 (1H, br), 8.41 (1H, br).	511.08<br>95.5%<br>(2.74 min)<br>Method B

20e was synthesized from corresponding *trans*-4-(4-methoxyphenyl)-2-methylcyclohexanecarbaldehyde.[8]

S19. Preparation of **20p** (route 1)

The synthesis procedure of typical compound **20p** was shown as below. Other compounds **20f, 20h-j** and **20q** were synthesized according to the same procedure as shown in the synthesis of compound **20p**.

Ethyl 4-(3-formyl-4-methoxyphenyl)-4-hydroxycyclohexane-1-carboxylate

1) A mixture of 2.01 g of 5-bromo-2-methoxybenzaldehyde, 2.0 ml of triethyl orthoformate, 207 mg of Amberlyst 15 hydrogen form and 10 ml of *n*-hexane was refluxed under an argon atmosphere for 19 hours. The reaction mixture was cooled to room temperature, filtered and evaporated under a vacuum to afford 4-bromo-2-(diethoxymethyl)-1-methoxybenzene 2.45g as a yellow oil.

2) To a solution of 2.45 g of 4-bromo-2-(diethoxymethyl)-1-methoxybenzene in 25 ml of dry tetrahydrofuran (THF), 3.22 ml of *n*-butyllithium (*n*-BuLi) in *n*-hexane solution (2.76 M) was added at -80°C and stirred for 2 hours under an argon atmosphere. The mixture was added dropwise onto 1.2 ml of ethyl 4-oxocyclohexane-1-carboxylate in 13 ml of dry THF solution at -80°C under an argon atmosphere and stirred for 19 hours at same temperature. The reaction mixture was quenched with saturated ammmonium chloride solution and extracted with AcOEt. The organic layer was washed with brine, dried over sodium sulfate, filtered and evaporated under a vacuum to afford 3.07 g of ethyl 4-[3-(diethoxymethyl)-4-methylphenyl]-4-hydroxycyclohexane-1-carboxylate as a brown oil.

3) A mixture of 3.07 g of ethyl 4-[3-(diethoxymethyl)-4-methylphenyl]-4-hydroxycyclohexane-1-carboxylate, 7.5 ml of 2N hydrochloric acid solution and 7.5 ml of THF was stirred at room temperature for 30 min. The reaction mixture was poured onto water, and extracted with AcOEt. The organic layer was washed with brine, dried over sodium sulfate, filtered and evaporated under a vacuum to afford a crude product that was purified by silica gel column chromatography to produce 606 mg of the title compound as a brown oil. MASS; m/z: 289.12 $(M-H_2O+H)^{+1}$, ^{1}H-NMR ($CDCl_3$) δ: 1.28 (3H, t, J=7.2 Hz), 1.55-1.65 (2H, m), 1.80-1.90 (2H, m), 1.92-1.98 (2H, m), 2.13-2.23 (1H, m), 2.30-2.50 (2H, m), 3.94 (3H, s), 4.14 (2H, q, J=7.2 Hz), 7.00 (1H, d, J=8.8 Hz), 7.77 (1H, dd, J=8.8, 2.8 Hz), 7.89 (1H, d, J=2.8 Hz), 10.50 (1H, s). Yield; q. y.

Ethyl 4-(3-cyano-4-methoxyphenyl)-4-hydroxycyclohexane-1-carboxylate (**22p**)

1) A mixture of 599 mg of ethyl 4-(3-formyl-4-methoxyphenyl)-4-hydroxycyclohexane-1-carboxylate, 163 mg of hydroxylamine hydrochloride, 5 ml of THF and 1 ml of H_2O was stirred at room temperature for 1.5 hours. The reaction mixture was quenched with water and extracted with AcOEt. The organic layer was washed with brine, dried over sodium sulfate, filtered and evaporated under a vacuum to afford a crude product that was purified by silica gel column chromatography to provide 601 mg of ethyl 4-hydroxy-4-{3-[(hydroxyamino)methyl]-4-methoxyphenyl}cyclohexane-1-carboxylate as a yellow oil. MASS m/z: 322.18 $(M+H)^+$

2) A mixture of 598 mg of ethyl 4-hydroxy-4-{3-[(hydroxyamino)methyl]-4-methoxyphenyl}cyclohexane-1-carboxylate, 0.78 ml of triethylamine and 672 mg of 2-chloro-1-methylpyridinium *p*-toluensulfonate was stirred at room temperature for 1 hour. The reaction

mixture was poured onto water, and extracted with AcOEt. The organic layer was washed with brine, dried over sodium sulfate, filtered and evaporated under a vacuum to afford a crude product that was purified by silica gel column chromatography to produce 464 mg of the title compound **22p** as a brown oil. Yield; 65.7 %.

Ethyl 4-allyl-4-(3-cyano-4-methoxyphenyl)cyclohexane-1-carboxylate (23p)

To a mixture of 650 mg of ethyl 4-(3-cyano-4-methoxyphenyl)-4-hydroxycyclohexane-1-carboxylate **22p** and 0.54 ml allyltrimethylsilane in 13 ml of dichloromethane, 0.35 ml of boron trifluoride ethyl ether complex was dropwise at -80°C under an argon atmosphere, and then the reaction mixture was stirred for 15 hours at room temperature. To the reaction mixture, saturated sodium chloride solution was added and extracted with dichloromethane. The organic layer was washed with brine, dried over sodium sulfate, filtered and evaporated under a vacuum to afford a crude product that was purified by silica gel column chromatography to afford 544 mg of the title compound **23p** as a yellow oil., MASS m/z: 328.22 $(M+H)^+$ Yield; q. y.

Ethyl 4-(3-cyano-4-methoxyphenyl)-4-(2,3-dihydroxypropyl)cyclohexane-1-carboxylate (24p)

To a mixture of 544 mg of ethyl 4-allyl-4-(3-cyano-4-methoxyphenyl)cyclohexane-1-carboxylate **23p**, 8 ml of acetonitrile and 2 ml of H_2O, 1.21 g of osmium oxide, immobilized catalyst I and 59 mg of *N*-methylmorpholine *N*-oxide were added in an ice bath, and the reaction mixture was stirred for 20 hours at room temperature. The reaction was filtered, and the filtrate was added with 20% sodium sulfite solution, and extracted with AcOEt. The organic layer was washed with brine, dried over sodium sulfate, filtered and evaporated under a vacuum to afford 598 mg of the title compound **24p** as a brown oil., MASS m/z: 362.20 $(M+H)^+$ Yield; q. y.

4-(3-Cyano-4-methoxyphenyl)-4-(2-oxoethyl)cyclohexane-1-carboxylate (25p)

To a mixture of 950 mg of ethyl 4-(3-cyano-4-methoxyphenyl)-4-(2,3-dihydroxypropyl)cyclohexane-1-carboxylate **24p**, 18 ml of THF and 9 ml of H_2O, 675 mg of sodium periodate was added in an ice bath, and the mixture was stirred at room temperature for 2 hours. The reaction mixture was quenched by water, and extracted with AcOEt. The organic layer was washed with brine, dried over sodium sulfate, filtered and evaporated under a vacuum to afford a crude product that was purified by silica gel column chromatography to produce 606 mg of the title compound **25p** as a yellow oil. MASS m/z: 330.20 $(M+H)^+$ Yield; 70.0%.

Ethyl 4-(2-bromoethyl)-4-(3-cyano-4-methoxyphenyl)cyclohexane-1-carboxylate (27p)

1) To a solution of 606 mg of 4-(3-cyano-4-methoxyphenyl)-4-(2-oxoethyl)cyclohexane-1-carboxylate in 12 ml of THF, 105 mg of sodium borohydride was added in an ice bath, and the reaction mixture was stirred at room temperature for 4 hours. The reaction mixture was quenched with saturated ammonium chloride solution and extracted with AcOEt. The organic layer was washed with brine, dried over sodium sulfate, filtered and evaporated under a vacuum to afford a crude ethyl 4-(3-cyano-4-methoxyphenyl)-4-(2-hydroxyethyl)cyclohexane-1-carboxylate **26p**.

2) To a mixture of crude ethyl 4-(3-cyano-4-methoxyphenyl)-4-(2-hydroxyethyl)cyclohexane-1-carboxylate **26p**, 910 mg of carbon tetrabromide and 6 ml of dichloromethane, 970 mg of

triphenylphosphine in 8 ml of dichloromethane solution was added in an ice bath, and the reaction mixture was stirred at room temperature for 1 hour. The solvent was distilled off under a vacuum, AcOEt was added. An insoluble residue was filtered off, the filtrate was evaporated under a vacuum, and the residue was purified by silica gel column chromatography to afford 575 mg of the title compound **27p** as a yellow oil. ^{1}H-NMR ($CDCl_3$) δ: 1.18-1.31 (3H, m), 1.35-1.58 (2H, m), 1.64-1.71 (1H, m), 1.79-1.91 (3H, m), 1.95-2.08 (2H, m), 2.20-2.27 (1H, m), 2.30-2.40 (2H, m), 2.90-3.02 (2H, m), 3.94 (3H, s), 4.02-4.40 (2H, m), 6.97 (1H, d, J=9.2 Hz), 7.40-7.50 (2H, m). Yield; 79.3 %.

Ethyl 4-(3-cyano-4-methoxyphenyl)bicyclo[2.2.2]octane-1-carboxylate (28p)

To a mixture of 1.24 ml of diisopropylamine and 4.4 ml of dry THF, 3.22 ml of *n*-BuLi in *n*-hexane solution (2.76 M) was added at 0°C under an argon atmosphere, and the mixture was stirred for 1 hour. The reaction mixture was added to a mixture of 575 mg of ethyl 4-(2-bromoethyl)-4-(3-cyano-4-methoxyphenyl)cyclohexane-1-carboxylate **27p** 575 mg, 1.52 ml of hexamethylphosphoric triamide and 25 ml of dry THF at -80°C under an argon atmosphere, and stirred for 4 hours. The reaction mixture was quenched with saturated ammonium chloride solution and extracted with AcOEt. The organic layer was washed with brine, dried over sodium sulfate, filtered and evaporated under a vacuum to afford a crude product that was purified by silica gel column chromatography to produce 259 mg of the title compound **28p** as a colorless solid. ^{1}H-NMR ($CDCl_3$) δ: 1.25 (3H, t, J=7.2 Hz), 1.79-1.94 (12H, m), 3.90 (3H, s), 4.12 (2H, q, J=7.2 Hz), 6.90 (1H, d, J=9.6 Hz), 7.46-7.49 (2H, m). Yield; 56.5 %.

5-[4-(Hydroxymethyl)bicyclo[2.2.2]octan-1-yl]-2-methoxybezonitrile (29p)

1) A mixture of 259 mg of ethyl 4-(3-cyano-4-methoxyphenyl)bicyclo[2.2.2]octane-1-carboxylate, 1.5 ml of 2N sodium hydroxide solution, 1.5 ml of ethanol and 1.5 ml of THF was stirred at 50°C for 15 hours. The reaction mixture was neutralized with 2N hydrochloric acid solution in an ice bath, and extracted with AcOEt. The organic layer was washed with brine, dried over sodium sulfate, filtered and evaporated under a vacuum to afford 234 mg of 4-(3-cyano-4-methoxyphenyl)bicyclo[2.2.2]octane-1-carboxylic acid as a colorless solid. ^{1}H-NMR ($CDCl_3$) δ: 1.81-1.96 (12H, m), 3.91 (3H, s), 6.90 (1H, d, J=10.0 Hz), 7.47-7.49 (2H, m). MASS m/z: 286.17 $(M+H)^+$.

2) To a solution of 174 mg of 4-(3-cyano-4-methoxyphenyl)bicyclo[2.2.2]octane-1-carboxylic acid in 2 ml of dichloromethane, 0.25 ml of *N*,*N*-diisopropylethylamine and 0.096 ml of isobutyl chloroformate were added in an ice bath, and the reaction mixture was stirred at room temperature for 3 hours. The solvent was evaporated under a vacuum. The residue in 4.0 ml of THF solution was added to a mixture of 69 mg of sodium borohydride, 1.0 ml of THF and 0.5 ml of H_2O in an ice bath. The reaction mixture was stirred at room temperature for 30 minutes. The reaction mixture was quenched with saturated ammonium chloride solution and extracted with AcOEt. The organic layer was washed with brine, dried over sodium sulfate, filtered and evaporated under a vacuum to afford 112 mg of the title compound **29p** as a colorless solid. ^{1}H-NMR ($CDCl_3$) δ: 1.55-1.85 (12H, m), 3.33 (2H, s), 3.90 (3H, s), 6.89 (1H, d, J=9.6 Hz), 7.47-7.49 (2H, m). Yield; 98.6 %.

5-(4-Formylbicyclo[2.2.2]octan-1-yl)-2-methoxybenzonitrile (30p)

To a mixture of 112 mg of 5-[4-(hydoxymethyl)bicyclo[2.2.2]octan-1-yl]-2-methoxybezonitrile **29p**, 0.3 ml of triethylamine, 0.15 ml of dimethyl sulfoxide and 2 ml of dichloromethane, 195 mg of pyridine sulfur trioxide complex was added in an ice bath, and the reaction mixture was stirred at room temperature for 8 hours. The reaction mixture was quenched with water and extracted with dichloromethane. The organic layer was washed with brine, dried over sodium sulfate, filtered and evaporated under a vacuum to afford a crude product that was purified by silica gel column chromatography to afford 84 mg of the title compound **29p** as a colorless solid. ^{1}H-NMR ($CDCl_3$) δ: 1.77-1.87 (12H, m), 3.91 (3H, s), 6.91 (1H, d, J=9.6Hz), 7.47-7.50 (2H, m), 9.52 (1H, s). MASS m/z:270.17 $(M+H)^+$ Yield; 56.2 %.

***N*-(3-Chloro-5-methoxyphenyl)-*N*-{[4-(3-cyano-4-methoxyphenyl)bicyclo[2,2,2]octan-1-yl]methyl}-3-methylisonicotinamide** (**20p**)

1) A mixture of 83 mg of 5-(4-formylbicyclo[2.2.2]octan-1-yl)-2-methoxybenzonitrile **30p**, 56 mg of 3-chloro-4-methoxyaniline, 106 mg of sodium triacetoxyborohydride, 36 mg of acetic acid and 2 ml of dichloromethane was stirred at room temperature for 1 hour, and the solvent was evaporated. The residue was dissolved in water. The aqueous layer was neutralized (pH=7) with 10% sodium hydroxide solution, and it was extracted with AcOEt. The organic layer was washed with brine, dried over sodium sulfate, filtered and concentrated under a vacuum to afford a crude product which was purified by silica gel column chromatography to yield 117 mg of 5-(4-{[(3-chloro-5-methoxyphenyl)amino]methyl}bicyclo[2.2.2]octan-1-yl)-2-methoxybenzonitrile as a brown amorphous. Yield; 23.7 %.

2) A mixture of 117 mg of 5-(4-{[(3-chloro-5-methoxyphenyl)amino]methyl} bicyclo[2.2.2]octan-1-yl)-2-methoxybenzonitrile, 165 mg of 3-methyl-4-pyridinecarbonyl chloride hydrochloride, 0.49 ml of *N*,*N*-diisopropylethylamine and 3 ml of dichloromethane was stirred at room temperature for 18 hours. The reaction mixture was quenched with water and extracted with dichloromethane. The organic layer was washed with brine, dried over sodium sulfate, filtered and evaporated under a vacuum to afford a crude product that was purified by silica gel column chromatography to afford 136 mg of the title compound **20p** as a pale-yellow solid. Yield; 85.9 %. ^{1}H-NMR($CDCl_3$) δ: 1.55-1.58 (6H, m), 1.73-1.76 (6H, m), 2.34 (3H, s), 3.66 (3H, s), 3.88-3.90 (5H, m), 6.41 (1H, m), 6.66 (2H, d, J=14.4 Hz), 6.82 (1H, d, J=4.8 Hz), 6.88-6.89 (1H, m), 7.43-7.45 (2H, m), 8.24 (1H, d, J=4.4 Hz), 8.38 (1H, s)., ^{13}C-NMR (DMSO-d_6) δ: 15.9, 29.7, 31.5, 33.9, 34.3, 55.8, 56.2, 57.1, 99.8, 111.9, 112.6, 113.2, 116.8, 119.7, 120.8, 128.7, 130.4, 132.4, 133.5, 142.5, 144.1, 145.2, 146.5, 150.9, 158.8, 159.8, 168.0., MASS m/z: 530.27 $(M+H)^+$, Purity 98.3% (R.T= 1.87 min) Method B. HRMS (ESI) calculated: 530.2205 $(M+H)^+$, found 530.2204.

S20. NMR and LCMS data of **20f**, **20h-j**, **20q**.

Supplementary Table S6. NMR and LCMS data of **20f**, **20h-j**, **20q**.

Ex.	Structure	NMR data	Mass m/z:$(M+H)^+$
**20f**	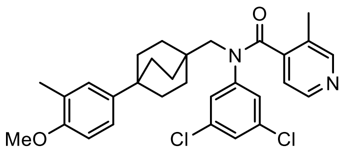	^{1}H-NMR ($CDCl_3$) δ: 1.42-1.64 (6H, m), 1.67-1.88 (6H, m), 2.20 (3H, s), 2.33 (3H, s), 3.80 (3H, s), 3.87 (2H, brs), 6.74 (1H, d, J=9.2 Hz), 6.77-6.87 (1H, m), 6.94 (2H, brs), 7.05 (1H, d, J=9.2 Hz), 7.06 (1H, s), 7.13 (1H, brs), 8.20-8.34 (1H, m), 8.41 (1H, brs)., ^{13}C-NMR (DMSO-d_6) δ: 15.9, 16.4, 29.9, 31.9, 33.5, 34.5, 55.1, 57.4, 109.7, 115.7, 121.0, 123.5, 124.7, 126.8, 127.7, 128.7, 133.8, 141.1, 143.6, 145.5, 146.6, 151.0, 155.2, 168.0.	523.16<br>95.1%<br>(2.71 min)<br>Method B
**20h**	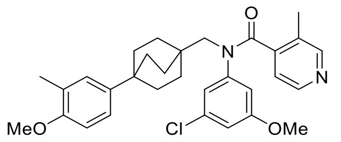	^{1}H-NMR ($CDCl_3$) δ: 1.42-1.67 (6H, m), 1.68-1.86 (6H, m), 2.19 (3H, s), 2.33 (3H, s), 3.65 (3H, s), 3.78 (3H, s), 3.85 (2H, brs), 6.43 (1H, brs), 6.64 (1H, brs), 6.69 (1H, brs), 6.72 (1H, d, J=9.2 Hz), 6.77-6.90 (1H, m), 7.03 (1H, d, J=9.2 Hz), 7.04 (1H, s), 8.15-8.31 (1H, m), 8.37 (1H, brs)., ^{13}C-NMR (DMSO-d_6) δ:16.0, 16.3, 29.9, 31.9, 33.5, 34.3, 55.1, 55.8, 57.2, 109.7, 112.6, 113.2, 119.7, 120.8, 123.5, 124.7, 127.7, 128.7, 133.5, 141.1, 144.1, 145.2, 146.5, 150.9, 155.2, 159.8, 168.0.	519.20<br>98.9%<br>(2.63 min)<br>Method B
**20i**	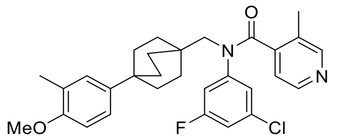	^{1}H-NMR ($CDCl_3$) δ: 1.42-1.67 (6H, m), 1.68-1.86 (6H, m), 2.19 (3H, s), 2.34 (3H, s), 3.80 (3H, s), 3.88 (2H, brs), 6.67 (1H, d, J=7.6 Hz), 6.74 (1H, d, J=9.2 Hz), 6.81 (1H, s), 6.87 (2H, s), 7.04 (1H, s), 7.06 (1H, s), 8.27 (1H, d, J=2.4 Hz), 8.41 (1H, brs).	507.21<br>97.2%<br>(2.52 min)<br>Method B
**20j**	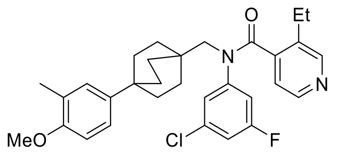	^{1}H-NMR ($CDCl_3$) δ: 1.32 (3H, t, J=7.2 Hz), 1.42-1.67 (6H, m), 1.68-1.86 (6H, m), 2.19 (3H, s), 2.68 (2H, q, J=7.6 Hz), 3.79 (3H, s), 3.87 (2H, brs), 6.67 (1H, d, J=8.0 Hz), 6.74 (1H, d, J=9.2 Hz), 6.79 (1H, s), 6.87 (2H, s), 7.04 (1H, s), 7.06 (1H, s), 8.27 (1H, m), 8.46 (1H, brs).	521.23<br>95.3%<br>(2.65 min)<br>Method B

Supplementary Table S6. NMR and LCMS data of **20f**, **20h-j**, **20q**. (continue)

Ex.	Structure	NMR data	Mass m/z:(M+H)$^+$
**20q**	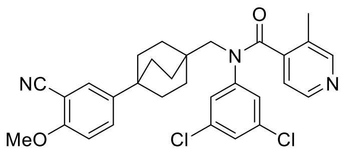	^{1}H-NMR ($CDCl_3$) δ: 1.53-1.58 (6H, m), 1.74-1.77 (6H, m), 2.33 (3H, s), 3.88-3.90 (5H, m), 6.81 (1H, brs), 6.87-6.90 (1H, m), 6.94 (2H, brs), 7.14 (1H, brs), 7.44-7.45 (2H, m), 8.29 (1H, brs), 8.41 (1H, s)., ^{13}C-NMR (DMSO-d_6) δ: 15.9, 29.6, 31.5, 33.8, 34.4, 56.2, 57.2, 99.8, 111.9, 116.7, 120.9, 126.7, 126.8, 128.7, 130.3, 132.3, 133.7, 142.4, 143.5, 145.5, 146.6, 151.0, 158.8, 168.0. HRMS (ESI) calculated: 534.17096 (M+H)$^+$, found 534.17096	534.28<br>95.4%<br>(2.10 min)<br>Method B

S21. Preparation of **30r** (route 2)

The synthesis procedure of typical compound **30r** was shown as below. Other compounds **30k-o**, **30s-v** were synthesized by same procedure of the synthesis of compound **30r**. **20k-o**, **20r-v** were synthesized from corresponding **30** (route 2) by the general procedure in the synthesis of compound **20p.**

Methyl 4-(hydroxymethyl)bicyclo[2.2.2]octane-1-carboxylate (**32**)

To a mixture of 500 mg of 4-(methoxycarbonyl)bicyclo[2.2.2]octane-1-carboxylic acid and 5 ml of dichloromethane, 0.24 ml of borane dimethyl sulfide complex was added in ice bath under an argon atmosphere. The reaction mixture was stirred for 3 hours at room temperature. The raction mixture was quenched with saturated sodium hydrogen carbonate solution and was extracted with AcOEt. The organic layer was washed with brine, dried over sodium sulfate, filtered and evaporated under a vacuum to afford a crude product that was purified by silica gel column chromatography to give 412 mg of the title compound **32** as a colorless oil. ^{1}H-NMR ($CDCl_3$) δ:1.41-1.51 (6H, m), 1.73-1.88 (6H, m), 3.29 (2H, s), 3.65 (3H, s). Yield; 88 %.

Methyl 4-[(2-methoxymethoxy)methyl]bicyclo[2.2.2]octane-1-carboxylate (**33**)

To a mixture of 412 mg of methyl 4-(hydroxymethyl)bicyclo[2.2.2]octane-1-carboxylate **32**, 1.2 ml of *N*,*N*-diisopropylethylamine and 5 ml of dichloromethane, 0.24 ml of chloromethylmethylether was added at 0°C under an argon atmosphere. The reaction mixture was stirred at room temperature for 15 hours. The reaction mixture was quenched with water and extracted with chloroform. The organic layer was washed with brine, dried over sodium sulfate, filtered and evaporated under a vacuum to afford a crude product that was purified by silica gel column chromatography to produce 497 mg of the title compound **33** as a colorless oil. ^{1}H-NMR ($CDCl_3$) δ: 1.43-1.52 (6H, m), 1.72-1.84 (6H, m), 3.16 (2H, s), 3.33 (3H, s), 3.64 (3H, s), 4.58 (2H, s). Yield; 99%.

4-[(2-Methoxymethoxy)methyl]bicyclo[2.2.2]octane-1-carboxylic acid (**34**)

A mixture of 497 mg of methyl 4-[(2-methoxymethoxy)methyl]bicyclo[2.2.2]octane-1-carboxylate **33**, 5 ml of methanol, 5 ml of THF and 10 ml of 2 M sodium hydroxide solution was stirred at room temperature for 15 hours. The reaction mixture was neutralized (pH=7) with 2N hydrochloric acid solution, and it was extracted with AcOEt. The organic layer was washed with brine, dried over sodium sulfate, and concentrated under a vacuum to afford 412 mg of the title compound **34** as a colorless oil. ^{1}H-NMR ($CDCl_3$) δ: 1.42-1.55 (6H, m), 1.75-1.88 (6H, m), 3.17 (2H, s), 3.35 (3H, s), 4.58 (2H, s). Yield; q. y.

1,3-Dioxoisoindolin-2-yl 4-[(2-methoxymethoxy)methyl]bicyclo[2.2.2]octane-1-carboxylate (35)

To a mixture of 460 mg of 4-[(2-methoxymethoxy)methyl]bicyclo[2.2.2]octane-1-carboxylic acid **34**, 334 mg of *N*-hydroxyphthalimide, 25 mg of *N*,*N*-dimethyl-4-aminopyridine and 10 ml of dichloromethane, 0.35 ml of *N*,*N'*-diisopropylcarbodiimide was added, and stirred at room temperature for 6 hours under an argon atmosphere. The reaction mixture was filtered through a Celite, and the solution was concentrated under a vacuum. The residue was purified by silica gel

column chromatography to produce 636 mg of the title compound **35** as a colorless solid. ^{1}H-NMR ($CDCl_3$) δ: 1.47-1.66 (6H, m), 1.94-2.10 (6H, m), 3.21 (2H, s), 3.35 (3H, s), 4.60 (2H, s), 7.73-7.82 (2H, m), 7.84-7.91 (2H, m). Yield; 83 %.

[(1,4-Benzodioxan-6-yl)bicyclo[2.2.2]octane-1-yl]methanol (**37r**)

1) To a mixture of 132 mg of dry magnesium, 192 mg of lithium chloride and 1ml of dry THF, 0.04 ml of diisobutylalminum hydride was added at room temperature under an argon atmosphere, and the mixture was stirred for 5 min.

2) To the above 1) mixture, 780 mg of 6-bromo-1,4-benzodioxane in 4 ml of THF was added at room temperature, and the mixture was stirred for 1 hour.

3) To a mixture of 427 mg of tris(2,4-pentanedionato)iron(III) and 3 ml of 1,3-dimethyl-3,4,5,6-tetrahydro-2(1*H*)-pyrimidinone, the above 2) solution was added at room temperature under an argon atmosphere, and the mixture was stirred for 2 hours. The reaction mixture was quenched by water and filtered through a Celite. The filtrate was extracted with AcOEt. The organic layer was washed with brine, dried over sodium sulfate, filtered and evaporated under a vacuum to afford a crude product that was purified by silica gel column chromatography to afford crude 6-{4-[(methoxymethoxy)methyl]bicyclo[2.2.2]octan-1-yl}-2,3-dihydrobenzo[b][1,4]dioxine.

4) A mixture of crude 6-{4-[(methoxymethoxy)methyl]bicyclo[2.2.2]octan-1-yl}-2,3-dihydrobenzo[b][1,4]dioxine and 10 ml of 2 M hydrogen chloride-dioxane solution was stirred at room temperature for 4 hours. The reaction mixture was quenched with water and extracted with AcOEt. The organic layer was washed with brine, dried over sodium sulfate, filtered and evaporated under a vacuum to afford a crude product that was purified by silica gel column chromatography to afford 106 mg of the title compound **37r** as a colorless oil. ^{1}H-NMR ($CDCl_3$) δ: 1.56-1.68 (6H, m), 1.76-1.83 (6H, m), 3.32 (2H, s), 4.23 (4H, d, J=1.0 Hz), 6.77-6.84 (3H, m). Yield; 32 %.

[(1, 4-Benzodioxan-6-yl)bicyclo[2.2.2]octane-1-yl]carbaldehyde (**30r**)

To a mixture of 106 mg of [(1, 4-benzodioxan-6-yl)bicyclo[2.2.2]octane-1-yl]methanol, 0.14 ml of dimethyl sulfoxide, 0.28 ml of triethylamine and 5 ml of dichloromethane 186 mg of pyridine sulfur trioxide complex was added in an ice bath under an argon atmosphere, and the reaction mixture was stirred at room temperature for 15 hours. The reaction mixture was quenched with water and extracted with chloroform. The organic layer was washed with brine, dried over sodium sulfate, filtered and evaporated under a vacuum to afford a crude product that was purified by silica gel column chromatography to afford 80 mg of the title compound **30r** as a colorless solid. ^{1}H-NMR ($CDCl_3$) δ: 1.71-1.89 (12H, m), 4.24 (4H, s), 6.78-6.83 (2H, m), 6.81-6.83 (1H, brs), 9.51 (1H, s). Yield; 76 %.

S22. NMR and LCMS data of **20k-o**, **20r-v**

Supplementary Table S7. NMR and LCMS data of **20k-o**, **20r-v**.

Ex.	Structure	NMR data	Mass m/z:(M+H)$^+$
**20k**	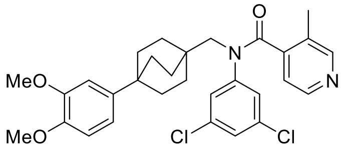	^{1}H-NMR ($CDCl_3$) δ: 1.50-1.65 (6H, m), 1.75-1.85 (6H, m), 2.34 (3H, s), 3.85 (3H, s), 3.87 (3H, s), 3.88 (2H, br), 6.78-6.83 (4H, m), 6.94 (2H, br), 7.13 (1H, br), 8.28 (1H, br), 8.41 (1H, br)., ^{13}C-NMR (DMSO-d_6) δ: 15.9, 29.9, 31.9, 33.9, 34.5, 55.4, 55.5, 57.4, 109.8, 111.5, 117.1, 120.9, 126.8, 128.8, 133.8, 142.2, 143.6, 145.6, 146.6, 146.7, 148.3, 151.1, 168.7, 126.8.	539.31 95.4% (2.08 min) Method B
**20l**	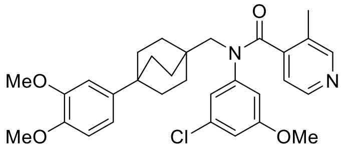	^{1}H-NMR ($CDCl_3$) δ: 1.46-1.69 (6H, m), 1.69-1.86 (6H, m), 2.34 (3H, s), 3.66 (3H, s), 3.85 (3H, s), 3.87 (3H, s), 3.88 (2H, brs), 6.42 (1H, brs), 6.64 (1H, brs), 6.69 (1H, brs), 6.80 (1H, s), 6.81 (2H, d, J=9.2 Hz), 6.82 (1H, brs), 8.25 (1H, brd, J=4.8 Hz), 8.38 (1H, brs).	535.30 96.5% (1.88 min) Method B
**20m**	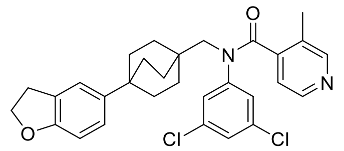	^{1}H-NMR ($CDCl_3$) δ: 1.45-1.63 (6H, m), 1.71-1.85 (6H, m), 2.33 (3H, s), 3.17 (2H, t, J=8.8 Hz), 3.87 (2H, s), 4.53 (2H, t, J=8.8 Hz), 6.70 (1H, d, J=8.4 Hz), 6.81 (1H, s), 6.94 (2H, s), 7.01 (1H, d, J=8.0 Hz), 7.12 (2H, s), 8.29 (1H, s), 8.41 (1H, s)., ^{13}C-NMR (DMSO-d_6) δ:15.9, 29.3, 29.9, 32.1, 33.8, 34.4, 57.4, 70.7, 108.1, 120.9, 122.1, 124.4, 126.7, 126.8, 128.6, 133.7, 141.6, 143.6, 145.5, 146.6, 151.0, 157.5, 168.0.	521.23 95.6% (2.36 min) Method B
**20n**	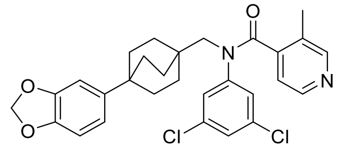	^{1}H-NMR($CDCl_3$) δ: 1.45-1.63 (6H, m), 1.69-1.83 (6H, m), 2.33 (3H, s), 3.87 (2H, s), 5.91 (2H, s), 6.72 (2H, s), 6.80 (2H, s), 6.94 (2H, s), 7.12 (1H, s), 8.28 (1H, s), 8.41 (1H, s). , ^{13}C-NMR (DMSO-d_6) δ:15.9, 29.8, 31.9, 34.2, 34.5, 57.3, 100.6, 106.4, 107.6, 117.9, 121.0, 126.7, 126.8, 128.7, 133.8, 143.6, 143.8, 144.9, 145.5, 146.6, 147.1, 151,0, 168.0.	523.21 95.1% (2.33 min) Method B
**20o**	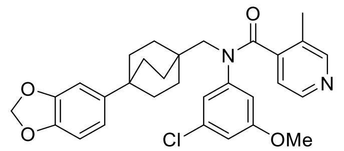	^{1}H-NMR($CDCl_3$) δ: 1.40-1.62 (6H, m), 1.71-1.81 (6H, m), 2.35 (3H, s), 3.67 (3H, s), 3.88 (2H, s) 5.91 (2H, s), 6.42 (1H, s), 6.67 (1H, s), 6.69 (1H, s), 6.72 (2H, s), 6.80 (1H, s), 6.83 (1H, d, J=4.8 Hz), 8.26 (1H, d, J=4.8 Hz), 8.38 (1H, s)., ^{13}C-NMR (DMSO-d_6) δ: 15.9, 29.8, 31.9, 34.2, 34.3, 55.8, 57.1, 100.5, 106.4, 107.6, 112.6, 113.1, 117.9, 119.7, 120.8, 128.7, 133.4, 143.8, 144.1, 144.9, 146.5, 147.1, 150.9, 159.8, 168.0.	519.25 95.2% (2.10 min) Method B

Supplementary Table S7. NMR and LCMS data of **20k-o**, **20r-v**. (continue)

Ex.	Structure	NMR data	Mass m/z:(M+H)$^+$
**20r**	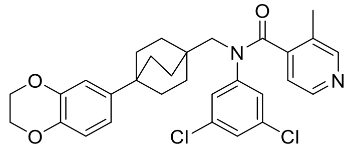	^{1}H-NMR($CDCl_3$) δ: 1.47-1.67 (6H, m), 1.71-1.81 (6H, m), 2.32 (3H, s), 3.86 (2H, s), 4.23 (4H, s), 6.71-6.84 (4H, m), 6.94 (2H, s), 7.12 (1H, s), 8.28 (1H, d, J=3.2 Hz), 8.40 (1H, brs)., ^{13}C-NMR (DMSO-d_6) δ: 15.9, 29.8, 31.8, 33.6, 34.4, 57.4, 63.9, 64.0, 114.1, 116.4, 118.0, 120.9, 126.7, 126.8, 128.7, 133.7, 141.1, 142.7, 143.6, 145.5, 146.6, 151.0, 168.0.	537.28<br>95.0%<br>(2.21 min)<br>Method B
**20s**	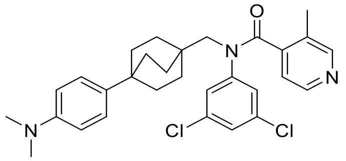	^{1}H-NMR ($CDCl_3$) δ: 1.47-1.65 (6H, m), 1.71-1.81 (6H, m), 2.34 (3H, s), 3.66 (3H, s), 3.69 (3H, s), 3.87 (2H, s), 6.41 (1H, s), 6.64 (1H, s), 6.68 (1H, s), 6.82 (1H, s), 6.90 (2H, s), 7.21-7.28 (2H, m), 8.25 (1H, d, J=4.8 Hz), 8.38 (1H, s).	522.39<br>95.0%<br>(2.40 min)<br>Method B
**20t**	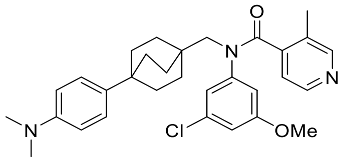	^{1}H-NMR ($CDCl_3$) δ: 1.47-1.65 (6H, m), 1.71-1.84 (6H, m), 2.34 (3H, s), 2.90 (6H, s), 3.65 (3H, s), 3.87 (2H, s), 6.41 (1H, s), 6.63 (1H, s), 6.67 (1H, s), 6.68 (2H, d, J=8.8 Hz), 6.82 (1H, d, J=4.8 Hz), 7.16 (2H, d, J=9.2 Hz), 8.25 (1H, d, J=4.4 Hz), 8.37 (1H, s).	518.43<br>97.0%<br>(1.17 min)<br>Method B
**20u**	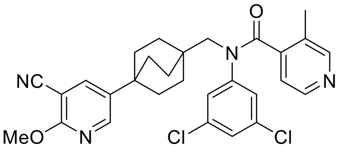	^{1}H-NMR ($CDCl_3$) δ: 1.47-1.65 (6H, m), 1.71-1.81 (6H, m), 2.33 (3H, s), 3.88 (2H, s), 3.92 (3H, s), 6.81 (1H, s), 6.94 (2H, s), 7.13 (1H, s), 7.31 (1H, s), 7.87 (1H, s), 8.28 (1H, s), 8.41 (1H, s).	524.37<br>95.0%<br>(2.31 min)<br>Method B
**20v**	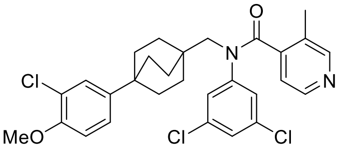	^{1}H-NMR ($CDCl_3$) δ: 1.47-1.63 (6H, m), 1.71-1.85 (6H, m), 2.33 (3H, s), 3.84-3.92 (5H, m), 6.81 (1H, s), 6.84 (1H, d, J=8.0 Hz), 6.93 (2H, s), 7.08-7.15 (2H, m), 7.22-7.31 (1H, m), 8.28 (1H, d, J=4.0 Hz), 8.41 (1H, s).	543.37<br>95.8%<br>(2.48 min)<br>Method B

S23. Preparation of **20g**

Ethyl 8-hydroxy-8-(4-methoxy-3-methylphenyl)bicyclo[3.2.1]octan-3-carboxylate

To a mixture of 760 mg of 4-bromo-1-methoxy-2-methylbenzene and 8 ml of dry THF, 1.37 ml of *n*-BuLi in *n*-hexane solution (2.76 M) was dropwise at -80°C under an argon atmosphere, and the mixture was stirred for 1 hour. The mixture was added dropwise onto 620 mg of ethyl 8-oxobicyclo[3.2.1]octan-3-carboxylate in 6 ml of THF solution at -80°C under an argon atmosphere and stirred for 4 hours at same temperature. The reaction mixture was quenched with saturated ammmoium chloride solution and extracted with AcOEt. The organic layer was washed with brine, dried over sodium sulfate, filtered and evaporated under a vacuum to afford a crude product that was purified by silica gel column chromatography to give 616 mg of the title compound as a yellow oil. MASS m/z: 301.20 $(M-H_2O+H)^+$ Yield; 68 %.

Ethyl 8-(4-methoxy-3-methylphenyl)bicyclo[3.2.1]octan-3-carboxylate

To a mixture of 819 mg of ethyl 8-hydroxy-8-(4-methoxy-3-methylphenyl)bicyclo[3.2.1]octan-3-carboxylate and 12 ml of dichloromethane, 0.82 ml of triethylsilane and 0.39 ml of trifluoroacetic acid were added at room temperature, and the mixture was stirred for 1 hour at room temperature. The reaction mixture was quenched with saturated sodium hydrogen carbonate solution and extracted with AcOEt. The organic layer was washed with brine, dried over sodium sulfate, filtered and evaporated under a vacuum to afford a crude product that was purified by silica gel column chromatography to produce 320 mg of the title compound as a colorless solid. MASS m/z: 302.99 $(M+H)^+$ Yield; 43 %.

8-(4-Methoxy-3-methylphenyl)bicyclo[3.2.1]octan-3-carbaldehyde

1) To a mixture of 320 mg of ethyl 8-(4-methoxy-3-methylphenyl)bicyclo[3.2.1]octan-3-carboxylate and 16 ml of dichloromethane, 2.7 ml of diisobutylalminum hydride in *n*-hexane solution (1.03 M) was added at -80°C under an argon atmosphere and stirred for 2 hours at room temperature. The reaction mixture was quenched with 10% potassium sodium tartrate solution, and the mixture was stirred for 16 hours. The mixture was extracted with dichloromethane. The organic layer was washed with brine, dried over sodium sulfate, filtered and evaporated under a vacuum to afford a crude [8-(4-methoxy-3-methylphenyl)bicyclo[3.2.1]octan-3-yl]methanol.

2) To a solution of [8-(4-methoxy-3-methylphenyl)bicyclo[3.2.1]octan-3-yl]methanol in 4 ml of dichloromethane, 0.38 ml of dimethyl sulfoxide, 0.74 ml of triethylamine and 506 mg of pyridine sulfur trioxide complex were added at room temperature, the reaction mixture was stirred for 1 hour. The raction mixture was quenched with water and extracted with dichloromethane. The organic layer was washed with brine, dried over sodium sulfate, filtered and evaporated under a vacuum to afford a crude product that was purified by silica gel column chromatography to give 180 mg of the title compound as a colorless solid. ^{1}H-NMR ($CDCl_3$) δ: 1.44 (2H, dd, J=15.2, 6.8 Hz), 1.74-1.90 (2H, m), 1.95-2.11 (5H, m), 2.24 (3H, s), 2.63 (2H, d, J=2.8 Hz), 2.87 (1H, t, J=4.0 Hz), 3.84 (3H, s), 6.82 (1H, d, J=8.4 Hz), 7.09 (1H, s), 7.10 (1H, d, J=8.4 Hz), 9.81 (1H, s). Yield; 66 %.

***N*-(3,5-Dichlorophenyl)-*N*-{[8-(4-methoxy-3-methylphenyl)bicyclo[3.2.1]octan-3-yl]methyl}-3-methylisonicotinamide (20g)**

The title compound was synthesized according to the synthesis procedure descibed in **13j** from 32 mg of 8-(4-methoxy-3-methylphenyl)bicyclo[3.2.1]octan-3-carbaldehyde, and afforded 25 mg as a pale brown amorphous. ^{1}H-NMR ($CDCl_3$) δ: 1.19-1.35 (2H, m), 1.37-1.51 (1H, br), 1.89-2.07 (6H, br), 2.20 (3H, s), 2.32 (3H, s), 2.62-2.74 (2H, br), 2.79-2.93 (1H, br), 3.80 (3H, s), 3.99 (2H, brd, J=8.0 Hz), 6.69-6.79 (3H, m), 6.82 (1H, brd, J=4.0 Hz), 6.96-7.15 (3H, m), 8.26 (1H, brd, J=4.0 Hz), 8.37 (1H, s). MASS m/z: 523.12$(M+H)^+$, purity; 95.3% (RT= 2.88 min, Method B) Yield; 39 % (2 steps).

S24. Supplementary Table S8. Structure activity relationships of 4-dimethylamino biphenyl derivatives

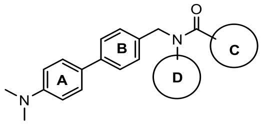

No	C	D	Efficacy (%) FXR (10 μM)	FXR (1 μM)	TGR5 (10 μM)	TGR5 (1 μM)
**13a**	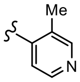	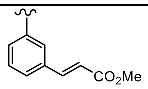	100.2	24.6	-3.1	N.T.
**13b**	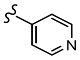	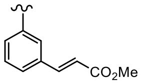	86.9	16.7	-2.8	N.T.
**13c**	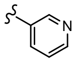	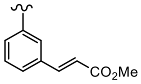	68.1	N.T.	1.7	N.T.
**13d**	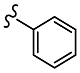	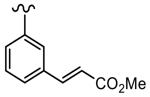	88.9	24.5	0.0	N.T.
**13e**	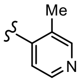	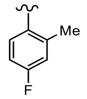	N.T.	2.0	87.5	N.T.
**13f**	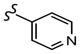	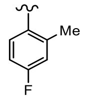	N.T.	0.5	32.0	N.T.
**13g**	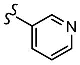	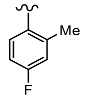	N.T.	1.2	0.8	N.T.
**13h**	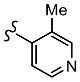	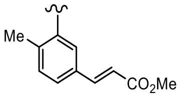	N.T.	25.3	0.2	N.T.
**13i**	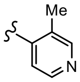	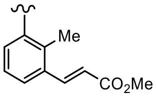	N.T.	32.1	47.2	N.T.
**13j**	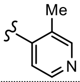	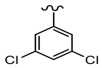	N.T.	62.1	59.7	N.T.
**7**			130.9	96.6 (1μM)<br>12.8 (0.1μM)	N.T.	N.T.
**8**			N.T.	N.T.	61.1	84.7

FXR; % vs OCA 1 μM as 100%, OCA; 100% (1 μM), 13.3% (0.1 μM); TGR5; % vs INT-767 10 μM as 100%, INT-767 100% (10 μM), 25.5% (1 μM), 1.7% (0.1 μM).

S25. Supplementary Table S9. Structure activity relationships of 4-dimethylaminobiphenylisonicotinamide derivatives

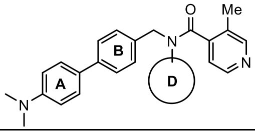

Compound	D	Efficacy (%) FXR (1 μM)	Efficacy (%) TGR5 (10 μM)
**13j**	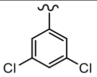	62.1	59.7
**13k**	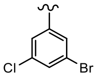	75.9	47.4
**13l**	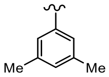	37.6	48.5
**13m**	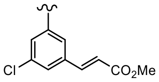	72.5	6.7
**13n**	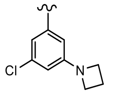	20.2	10.2
**13o**	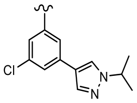	68.8	-1.5
**13p**	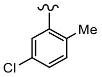	1.9	93.4
**13q**	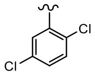	1.5	75.2
**13r**	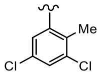	8.6	91.9
**13s**	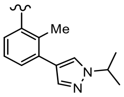	61.0	24.1
**13t**	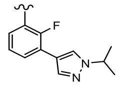	92.6	8.3
**13u**	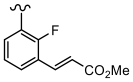	90.3	-2.5
**7**		96.6 (1μM)<br>12.8 (0.1μM)	N.T.
**8**		N.T.	66.1 (1μM)<br>84.7 (0.1μM)

FXR; % vs OCA 1 μM as 100%, OCA; 100% (1 μM), 13.3% (0.1 μM); TGR5; % vs INT-767 10 μM as 100%, INT-767 100% (10 μM), 25.5% (1 μM), 1.7% (0.1 μM).

S26. Supplementary Table S10. Structure activity relationships of 4-dimethylaminobiphenyl derivatives

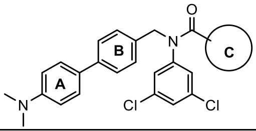

No	C	Efficacy (%)	
		FXR (1 μM)	TGR5 (10 μM)
**13j**	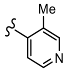	62.1	59.7
**13v**	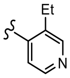	21.3	65.3
**13w**	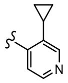	22.7	73.1
**13x**	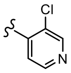	62.1	9.8
**13y**	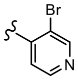	51.6	-2.0
**13z**	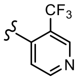	10.6	24.4
**13aa**	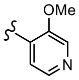	68.6	21.5
**13ab**	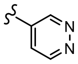	0.5	-12
**13ac**	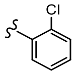	84.3	0.2
**13ad**	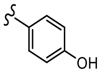	88.2	4.4
**13ae**	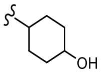	101.4	-1.5
**13af**	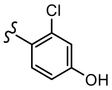	108.2	-0.2
**13ag**	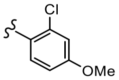	136.5	1.1
**7**		96.6 (1μM)<br>12.8 (0.1μM)	N.T.
**8**		N.T.	66.1 (1μM)<br>84.7 (0.1μM)

FXR; % vs OCA 1 μM as 100%, OCA; 100% (1 μM), 13.3% (0.1 μM); TGR5; % vs INT-767 10 μM as 100%, INT-767 100% (10 μM), 25.5% (1 μM), 1.7% (0.1 μM).

S27. Supplementary Table S11. Structure activity relationships of isonicotinamide derivatives

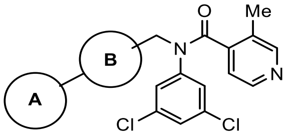

No	A	B	Efficacy (%) FXR (10 µM)	FXR (1 µM)	TGR5 (10 µM)
**13j**	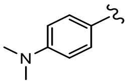	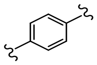	N.T.	62.1	59.7
**13ah**	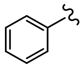	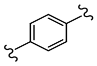	N.T.	17.7	118.6
**20a**	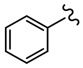	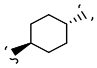	N.T.	52.2	22.3
**20b**	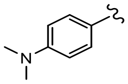	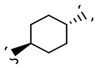	106.8	47.1	31.1
**20c**	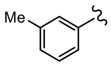	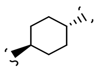	N.T.	51.7	5.7
**20d**	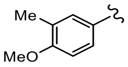	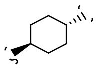	129.6	55.2	28.8
**20e**	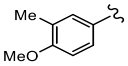	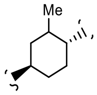	72.6	69.1	33.9
**20f**	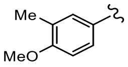	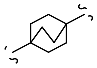	64.3	90.3	28.8
**20g**	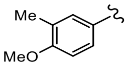	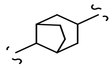	N.T.	51.1	-5.2
**7**			N.T.	96.6 (1µM)<br>12.8 (0.1µM)	N.T.
**8**			N.T.	N.T.	66.1 (1µM)<br>84.7 (0.1µM)

FXR; % vs OCA 1 µM as 100%, OCA; 100% (1 µM), 13.3% (0.1 µM); TGR5; % vs INT-767 10 µM as 100%, INT-767 100% (10 µM), 25.5% (1 µM), 1.7% (0.1 µM).

S28. Supplementary Table S12. Structure activity relationships of bicyclo[2.2.2]octane-isonicotinamide derivatives

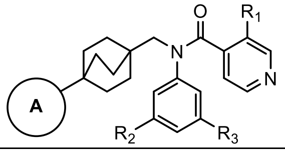

No	A	R_1	R_2	R_3	Efficacy (%)			
					FXR		TGR5	
					(1 µM)	(0.1 µM)	(10 µM)	(1 µM)
**20f**	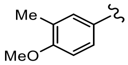	Me	Cl	Cl	90.3	N.T.	28.8	N.T.
**20h**	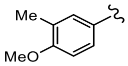	Me	Cl	OMe	89.8	34.4	73.6	21.0
**20i**	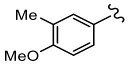	Me	Cl	F	89.2	62.6	62.1	N.T.
**20j**	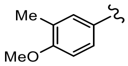	Et	Cl	F	71.7	13.5	71.1	21.5
**20k**	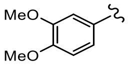	Me	Cl	Cl	75.7	35.0	88.6	N.T.
**20l**	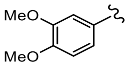	Me	Cl	OMe	59.9	3.6	52.0	23.1
**20m**	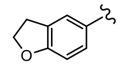	Me	Cl	Cl	91.5	58.5	72.4	N.T.
**20n**	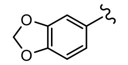	Me	Cl	Cl	98.9	73.5	67.6	N.T.
**20o**	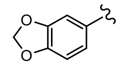	Me	Cl	OMe	97.6	29.4	78.9	36.0
**20p**	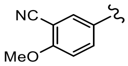	Me	Cl	OMe	98.8	57.1	62.4	51.1
**20q**	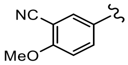	Me	Cl	Cl	79.6	85.7	80.2	51.2
**20r**	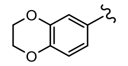	Me	Cl	Cl	72.6	28.0	N.T.	57.1
**20s**	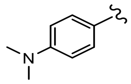	Me	Cl	Cl	95.3	56.4	N.T.	18.9
**20t**	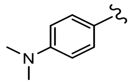	Me	Cl	OMe	77.3	32.8	N.T.	25.0
**20u**	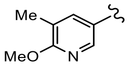	Me	Cl	Cl	76.2	69.2	N.T.	12.1
**20v**	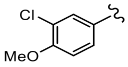	Me	Cl	Cl	79.6	65.4	N.T.	33.5
**7**					96.6	12.8	N.T.	N.T.
**8**					N.T.	N.T.	N.T.	66.1 (1µM)<br>84.7 (0.1µM)

FXR; % vs OCA 1 µM as 100%, OCA; 100% (1 µM), 13.3% (0.1 µM); TGR5; % vs INT-767 10 µM as 100%, INT-767 100% (10 µM), 25.5% (1 µM), 1.7% (0.1 µM).

S29. Docking and computational method.

Molecular docking between compound **20p** and FXR were performed by Discovery Studio 2017 software (Dassault Systems Biovia). FXR protein was download from Protein Data Bank (PDB) ID: 1OSH. The protein receptor was used for docking all compounds after undergoing the protein preparation process, such as supplementing amino acid residues and adding hydrogen atoms. The docking ligands (CDOCKER) protocol, which is a grid-based molecular docking method that employs CHARMm, was used in this study. compound 7 was first re-docked to the binding site. Consequently, all compounds were docked into the same active site, and then ten conformations of each compounds were obtained through CDOCKER. The highest reasonable docking pose was used.

S30. Reference

1. https://indigobiosciences.com/indigo-kits-services/farnesoid-x-receptor-fxr-nr1h4/, Indigo biosciences, Human FXR Reporter Assay System, 1 x 96-well format assays kit.
2. https://discoverx.com/home
3. Kemmer, G. & Keller, S. Nonlinear least-squares data fitting in Excel spreadsheets. *Nature Protocol* **5**, 267–281 (2010).
4. Matsumoto, M. *et al.* An improved mouse model that rapidly develops fibrosis in non-alcoholic steatohepatitis. *Int. J. Exp. Pathol*. **94**, 93–103 (2013).
5. Duan, H. *et al*. Design, synthesis, and antidiabetic activity of 4-phenoxynicotinamide and 4-phenoxypyrimidine-5-carboxamide derivatives as potent and orally efficacious TGR5 agonists. *J. Med. Chem.* **55**, 10475-10489 (2012).
6. Smith. N. D., Govek, S. P. & Nagasawa. J. Y. Farnesoid X Receptor Agonist and Uses Thereof. WO2017/049172, 23, March (2017).
7. Smith. N. D., Govek, S. P. & Nagasawa. J. Y. Farnesoid X Receptor Agonist and Uses Thereof. WO2017/049173, 23, March (2017).
8. Smith, N. D., Govek, S. P., Nagasawa, J. Y. & Douglas, K. L.; Farnesoid X Receptor Agonist and Uses Thereof. WO2018/170165, 20, September (2018).
